# Smart nanodelivery systems targeting tumor-associated macrophage reprogramming to potentiate cancer immunotherapy: pharmacological mechanisms, delivery strategies and translational perspectives

**DOI:** 10.3389/fphar.2026.1919263

**Published:** 2026-09-23

**Authors:** Xian Sun, Chenchen Chu, Jiajie Wang

**Affiliations:** 1 School of Chemistry, University of Glasgow, Glasgow, United Kingdom; 2 Taizhou Data Industry Group Co., Ltd., Taizhou, Jiangsu, China; 3 Department of Public Administration and Policy, School of Government, University of Birmingham, Birmingham, United Kingdom

**Keywords:** cancer immunotherapy, immune checkpoint blockade, macrophage reprogramming, nanomedicine, smart nanodelivery, translational pharmacology, tumor microenvironment, tumor-associated macrophages

## Abstract

Cancer immunotherapy, particularly immune checkpoint blockade, has substantially changed the treatment landscape for multiple malignancies, but clinical benefit remains limited by low response rates, primary tolerance, and acquired resistance. Tumor-associated macrophages (TAMs) are abundant and highly plastic myeloid cells in the tumor immune microenvironment. They contribute to tumor progression and immunotherapy resistance by suppressing T-cell recruitment and effector function, promoting angiogenesis, remodeling extracellular matrix, regulating metabolism, and expressing immune-checkpoint-related molecules. TAMs exhibit overlapping functional states beyond a fixed M1/M2 dichotomy, shaped by cytokines, chemokines, pattern-recognition receptors, kinase-transcription factor networks, hypoxia, lactate, and redox signals. Reprogramming immunosuppressive TAMs toward inflammatory, antigen-presenting, and T-cell-supporting phenotypes has therefore become an important pharmacological strategy to enhance antitumor immunotherapy. Smart nanodelivery systems can improve the *in vivo* stability, tumor accumulation, TAM exposure, and spatiotemporal release of immunomodulators, small-molecule inhibitors, nucleic acid therapeutics, cytokines, and macrophage immune-checkpoint modulators, thereby improving efficacy while reducing systemic toxicity. This review summarizes the rationale for targeting TAMs in cancer immunotherapy, the mechanisms regulating TAM functional states, the design principles of smart nanodelivery systems, nanomedicine-mediated reprogramming strategies, pharmacodynamic outcomes, pharmacokinetic and safety issues, preclinical evaluation methods, translational barriers, and future directions.

## 1 Introduction

Immune checkpoint blockade, chimeric antigen receptor cell therapy, and cancer vaccines have shifted the therapeutic paradigm for malignant tumors. However, except for a limited number of highly immunogenic cancers, many patients with solid tumors still show modest responses to anti-PD-1, anti-PD-L1, or anti-CTLA-4 therapy, and some patients who initially benefit ultimately develop acquired resistance (Ribas and Wolchok, 2018). Accumulating evidence indicates that treatment failure is not determined only by tumor-cell-intrinsic mutations but also by dynamic interactions among myeloid cells, lymphocytes, vasculature, stromal components, and metabolic states within the tumor immune microenvironment (TIME) (Binnewies et al., 2018; Yang et al., 2023).

Tumor-associated macrophages (TAMs) are among the most abundant innate immune cells in the TIME. They are frequently enriched in solid tumors and support tumor progression by suppressing cytotoxic T cells, inducing regulatory T cells, secreting IL-10 and TGF-β, promoting angiogenesis, remodeling extracellular matrix, and contributing to pre-metastatic niche formation (Qian and Pollard, 2010; DeNardo and Ruffell, 2019). In contrast to therapies that directly kill tumor cells, TAM-targeted intervention aims to remodel the tumor ecosystem so that the immune system can regain the capacity to recognize and eliminate malignant cells (Mantovani et al., 2022).

The pharmacological appeal of TAMs arises from their high plasticity. Macrophages can shift among inflammatory, antigen-presenting, immunosuppressive, tissue-repair, pro-angiogenic, and phagocytic states in response to microenvironmental signals. Although the conventional M1/M2 model remains useful for conceptualizing polarization (Murray et al., 2014; Locati et al., 2020), TAMs in real tumors do not exist as two fixed subtypes. Instead, they form a continuum of functional states that depends on spatial position, tumor type, and treatment history (Pittet et al., 2022). Therefore, TAM reprogramming should not be understood as a simple conversion of all M2-like TAMs into M1-like macrophages; rather, it should be defined as a pharmacological process that suppresses immunosuppressive and tumor-promoting programs while enhancing antigen presentation, inflammatory activation, and T-cell-supporting functions.

Direct administration of immunomodulatory agents faces several limitations. TLR and STING agonists can strongly activate innate immunity, but systemic exposure may cause inflammatory toxicity. Cytokines possess potent immune-stimulatory activity but are limited by short half-lives and dose-limiting toxicity. Nucleic acid therapeutics can regulate gene expression precisely, but naked nucleic acids are susceptible to degradation and inefficient membrane transport. Small-molecule pathway inhibitors may suffer from insufficient tumor exposure and off-target effects in normal tissues. Smart nanodelivery systems improve drug stability, prolong circulation, enhance tumor accumulation, enable active TAM targeting, and provide tumor-microenvironment-responsive release (Goldberg, 2019; Irvine and Dane, 2020), thereby offering a more controllable pharmacological platform for TAM reprogramming (Martin et al., 2020).

This narrative review was prepared by searching PubMed, Web of Science, Scopus, and Google Scholar using terms including tumor-associated macrophages, macrophage polarization, TAM reprogramming, nanomedicine, smart drug delivery, cancer immunotherapy, immune checkpoint blockade, TLR agonist, STING agonist, PI3Kγ, and CD47/SIRPα. The review prioritizes high-impact reviews, mechanistic studies, and representative preclinical nanomedicine studies published within the past decade, while retaining foundational publications in the field.

Recent advances have substantially updated the conceptual basis of TAM research. Earlier studies often regarded TAMs mainly as M2-like immunosuppressive cells, whereas single-cell RNA sequencing, spatial transcriptomics, and intravital imaging have shown that TAMs display continuous functional states across cancer types, spatial niches, and treatment stages. Immunosuppression, antigen presentation, angiogenesis, matrix remodeling, and metabolic regulation can coexist within the same tumor (Cassetta et al., 2019; Ma et al., 2022; Cassetta and Pollard, 2023; Cao and Liu, 2024). This conceptual shift has moved TAM-targeted therapy away from broad depletion and toward functional reprogramming and subtype-selective regulation.

In parallel, nanomedicine has evolved from passive delivery based mainly on the enhanced permeability and retention effect toward rational designs that account for protein corona formation, liver and spleen clearance, tissue penetration, immune-cell uptake, and microenvironment-responsive release. Because TAMs belong to the mononuclear phagocyte system, their natural uptake of nanoparticles can lead to off-target capture in liver and spleen, but it can also be exploited to increase the intratumoral exposure of immunomodulatory agents in TAMs (Wilhelm et al., 2016; Tsoi et al., 2016; van der Meel et al., 2019; Zhang et al., 2024). Thus, integrating TAM plasticity with smart nanodelivery represents an important intersection between tumor immunopharmacology and materials-based drug delivery.

This review discusses TAMs as pharmacological targets, the signaling mechanisms regulating TAM functional states, design principles for smart nanodelivery systems, nanomedicine-mediated TAM reprogramming strategies, pharmacodynamic consequences, pharmacokinetic and safety considerations, preclinical evaluation methods, translational bottlenecks, and future directions.

## 2 Tumor-associated macrophages as pharmacological targets in cancer immunotherapy

TAMs serve as central nodes linking inflammation, metabolism, matrix remodeling, and immune escape within the tumor microenvironment. Treating TAMs as pharmacological targets means that therapy is not restricted to eliminating a cell population; instead, the goal is to reshape tumor immune ecology by regulating TAM recruitment, survival, functional state, phagocytosis, and intercellular communication (Cassetta and Pollard, 2018; Pathria et al., 2019). Research on CSF-1R, CCR2, PI3Kγ, and CD47/SIRPα has shown that TAM-associated pathways are druggable and can cooperate with immune checkpoint blockade (Mantovani et al., 2022).

The recent trajectory of cancer immunotherapy research shows that explanations for treatment failure have expanded from tumor mutational burden, antigen expression, and T-cell exhaustion to myeloid-cell-mediated immune exclusion and drug-exposure control. TAMs are central in this transition because they can alter the spatial distribution of T cells (Peranzoni et al., 2018), influence antibody disposition through Fc-receptor-dependent capture (Arlauckas et al., 2017), regulate antigen presentation, and shape cytokine networks that determine the efficacy of immune checkpoint blockade (Yang et al., 2023).

Nanodelivery research has undergone a similar conceptual shift. Early studies emphasized passive nanoparticle accumulation in tumors, whereas later work increasingly focused on stromal penetration, mononuclear phagocyte system clearance, protein-corona-induced identity changes, and intracellular delivery efficiency in immune cells (Wilhelm et al., 2016; van der Meel et al., 2019). Consequently, TAM-targeted nanomedicine is no longer only a carrier problem but a strategy that connects pharmacokinetics, immunopharmacology, and tumor-ecosystem regulation (Martin et al., 2020).

### 2.1 Origin, recruitment, and heterogeneity of TAMs

TAMs arise mainly from two sources. One source is circulating monocytes that enter tumor tissue under the influence of chemokine and growth-factor signals such as CCL2/CCR2, CSF-1/CSF-1R, VEGF, and CXCL12/CXCR4 and then differentiate locally. The other source is tissue-resident macrophages that expand and undergo functional remodeling during tumorigenesis. Macrophages of different developmental origins have distinct histories, transcriptional programs, and metabolic preferences, producing highly heterogeneous TAM populations in tumors (Qian and Pollard, 2010; Noy and Pollard, 2014; DeNardo and Ruffell, 2019).

TAM heterogeneity is expressed across cancer type, spatial niche, and therapeutic pressure. The composition of TAMs differs among pancreatic cancer, breast cancer, glioma, hepatocellular carcinoma, and lung cancer, and these differences are shaped by organ-specific microenvironments and tumor evolution. Within a single tumor, TAMs near blood vessels, hypoxic necrotic regions, invasive margins, and lymphocyte-enriched areas may exhibit different transcriptional features (Pittet et al., 2022; Christofides et al., 2022). Therapy itself can also alter TAM states, because chemotherapy, radiotherapy, anti-angiogenic therapy, and immunotherapy may induce compensatory myeloid remodeling. Single-cell RNA sequencing, spatial transcriptomics, and multiplex immunofluorescence have therefore moved TAM research from bulk quantification toward spatial and functional subtype analysis (Qian et al., 2024).

The understanding of TAM origin has also changed. Earlier views emphasized continuous recruitment from circulating monocytes, but developmental immunology has shown that many tissue-resident macrophages arise from embryonic precursors and persist locally. Consequently, macrophage origin within tumors is organ-dependent and stage-dependent (Ginhoux and Guilliams, 2016; Laviron and Boissonnas, 2019). In pancreatic cancer, brain tumors, liver cancer, and other organ-specific tumors, tissue-resident macrophages and monocyte-derived macrophages frequently differ in transcriptional state, spatial localization, and treatment response (Ma et al., 2022; Cassetta and Pollard, 2023). Blocking monocyte entry alone may therefore fail to cover all tumor-promoting TAM subtypes.

Studies of human tumor samples have further deepened the understanding of TAM heterogeneity. Cassetta and colleagues generated monocyte and TAM transcriptional maps from human breast, endometrial, and ovarian cancer samples and showed that TAMs in different cancers do not share a fixed universal program. Instead, they exhibit cancer-type-associated reprogramming, prognostic signatures, and potential therapeutic targets (Cassetta et al., 2019). This observation implies that TAM-targeted nanodelivery should be tailored to tumor type and patient-specific TAM states rather than assuming that all CD206-positive or CD163-positive cells have the same pharmacological meaning (Pittet et al., 2022; Ma et al., 2022; He et al., 2023; He et al., 2024).

### 2.2 Functional states of TAMs: from M1/M2 model to dynamic states

TAMs exhibit heterogeneous, dynamic, and overlapping inflammatory, antigen-presenting, immunosuppressive, tissue-repair, angiogenic, and metabolic programs that vary with tissue context and treatment (Cassetta and Pollard, 2023; Ma et al., 2022). The M1/M2 terminology describes simplified experimental activation conditions rather than distinct TAM populations. Classically activated M1-like macrophages, typically induced by IFN-γ with microbial stimuli such as LPS, show inflammatory and antigen-presenting features; IL-4- or IL-13-stimulated M2-like macrophages show alternative activation and tissue-repair features (Murray et al., 2014; Locati et al., 2020). IL-10, TGF-β, lactate, and hypoxia can shape additional, overlapping tumor-associated programs. CD80, CD86, MHC-II, iNOS, CD206, CD163, and Arg-1 are context-dependent descriptors and do not define mutually exclusive or uniformly antitumor versus tumor-promoting states.

Throughout this review, TAM functional state remodeling denotes changes in macrophage programs, whereas TAM reprogramming denotes an intervention-associated functional change that reduces tumor-promoting activity or strengthens antitumor immune support. M1-like/M2-like and polarization are retained as qualified descriptions of experimental stimulation or reported phenotypes. A single TAM may express both M1-and M2-associated markers, and CD206-positive or CD163-positive cells may retain antigen-presenting capacity under certain conditions. Increased CD86 or decreased CD206 alone does not establish functional reprogramming. Therefore, evaluation of TAM reprogramming should combine phenotypic, transcriptional, cytokine, phagocytic, antigen-presentation, and T-cell activation readouts.

The M1/M2 model has operational clarity in simplified *in vitro* cytokine stimulation systems, but it cannot capture the simultaneous presence of hypoxia, lactate, cell death, matrix stiffness, and treatment pressure in tumor tissue. Single-cell and spatial omics studies indicate that TAMs more often show combined inflammatory, angiogenic, lipid-metabolic, phagocytic, antigen-presenting, and tissue-repair programs (Ma et al., 2022), rather than two endpoints along a single axis (Murray et al., 2014; Locati et al., 2020). Recent single-cell studies have further identified metabolically specialized TAM states, including lipid-associated macrophages (LAMs), that are not adequately represented by the conventional M1/M2 axis. In triple-negative breast cancer, FIG4 downregulation was reported to impair autophagy–lysosomal degradation of IL-18, thereby increasing IL-18 secretion, promoting LAM polarization and infiltration, and contributing to resistance to PD-1 blockade. Restoration of FIG4 expression or IL-18 inhibition resensitized tumors to immunotherapy, highlighting lipid metabolism and cytokine degradation as additional mechanisms of macrophage-mediated immune resistance (Xia et al., 2026).

A more reliable approach is to integrate phenotypic markers with functional endpoints. CD80, CD86, MHC-II, CD206, and CD163 can provide initial evidence of state changes, but cytokine profiles, phagocytosis indices, antigen-presentation capacity, antigen-dependent T-cell co-culture responses, and spatial proximity analyses are needed to determine whether a drug has actually reduced tumor-promoting functions and strengthened antitumor immune support (Cassetta and Pollard, 2023; Rannikko and Hollmén, 2024).

### 2.3 TAM-mediated resistance to cancer immunotherapy

TAM-mediated resistance to immunotherapy involves interconnected processes including T-cell suppression, construction of an immunosuppressive ecology, and regulation of checkpoint signals. Immunosuppressive TAMs act through IL-10, TGF-β, PGE2, and arginase-associated metabolism, thereby reducing CD8^+^ T-cell proliferation, cytotoxicity, and IFN-γ production and promoting T-cell exhaustion. TAMs can also recruit Treg cells through chemokines such as CCL22, impede T-cell entry through angiogenesis and matrix remodeling, and form suppressive networks with myeloid-derived suppressor cells, cancer-associated fibroblasts, and endothelial cells. Some TAMs express PD-L1, VISTA, and B7-H4, whereas tumor-cell CD47 engages macrophage SIRPα to inhibit phagocytosis (DeNardo and Ruffell, 2019; Mantovani et al., 2022; Xu et al., 2023). Figure 1 integrates these mechanisms into a TAM-centered view of immunotherapy resistance.

**FIGURE 1:**
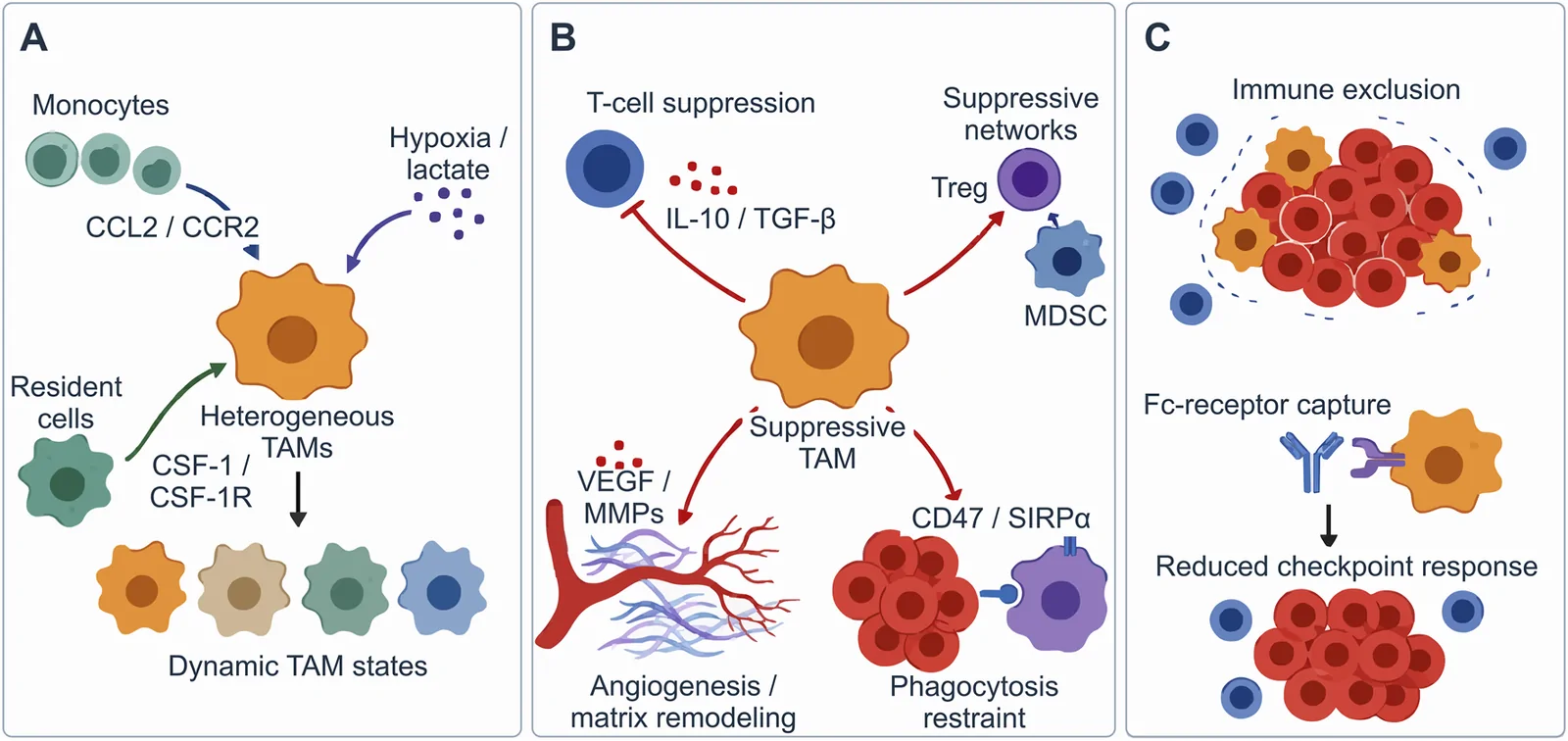
TAM-mediated immunosuppression and resistance to immune checkpoint blockade. **(A)** Monocyte recruitment and tissue-resident macrophages contribute to dynamic TAM states shaped by local signals. **(B)** Tumor-supporting TAM functions include T-cell suppression, suppressive-cell networks, angiogenesis and matrix remodeling, and phagocytosis restraint. **(C)** Immune exclusion and Fc-receptor-associated antibody capture can reduce checkpoint-treatment activity. The schematic summarizes context-dependent mechanisms and does not assign fixed functions to M1/M2 categories. TAM, tumor-associated macrophage; Treg, regulatory T cell; MDSC, myeloid-derived suppressor cell; MMP, matrix metalloproteinase.

Thus, TAMs are not bystanders in immunotherapy but regulators of whether a tumor behaves as an immunologically cold or hot lesion. Reprogramming TAMs from immunosuppressive and tumor-promoting states into inflammatory and antigen-presenting states may increase CD8^+^ T-cell infiltration, improve anti-PD-1/PD-L1 efficacy, and reduce recurrence and metastasis risk.

Intravital imaging has provided direct evidence that TAMs can mediate resistance to immunotherapy. Arlauckas and colleagues observed that anti-PD-1 antibodies initially bound tumor-infiltrating CD8^+^ T cells after administration, but were subsequently captured from the T-cell surface by PD-1-negative TAMs through an Fc-receptor-associated mechanism, potentially reducing sustained antibody occupancy on target T cells (Arlauckas et al., 2017). This finding extends the role of TAMs from producers of suppressive mediators to regulators of the *in vivo* fate of antibody drugs and suggests that nanodelivery or antibody engineering should consider myeloid-cell effects on biologic distribution.

Spatial studies further show that TAMs can restrict CD8^+^ T cells from entering tumor-cell nests, creating an immune-excluded phenotype and weakening anti-PD-1 responses (Peranzoni et al., 2018). In addition, PD-1 can be expressed by some TAMs and suppress macrophage phagocytosis (Gordon et al., 2017). Phagocytosis checkpoints such as CD24/Siglec-10 and CD47/SIRPα further demonstrate that TAM-mediated immune escape involves both T-cell suppression and phagocytosis inhibition (Barkal et al., 2019; Feng et al., 2019).

## 3 Pharmacological mechanisms regulating TAM functional states

TAM functional states are regulated by multiple pharmacologically tractable layers, including cytokine and chemokine networks, pattern-recognition receptors, kinase and transcription-factor pathways, metabolic reprogramming, and redox status. For readers in pharmacology, the key point is that nanodelivery systems are not themselves the final therapeutic targets. Therapeutic efficacy is determined by how the delivered cargo modulates TAM signaling networks and functional states.

The development of TAM polarization research reflects a transition from single-stimulus and single-phenotype models to multi-signal network models. Classical cytokines such as IFN-γ, IL-4, and IL-13 remain fundamental for understanding macrophage activation (Murray et al., 2014; Locati et al., 2020), but actual TAM states in tumors are shaped jointly by chemokine recruitment, receptor activation, metabolic stress, hypoxia, cell death, and therapy-induced inflammation (Cassetta and Pollard, 2023).

This network character means that pharmacological intervention must consider pathway sequence and feedback regulation. TLR or STING activation can enhance inflammation and antigen presentation, but high levels of IL-10, TGF-β, lactate, and adenosine may still drive TAMs back toward immunosuppression (Colegio et al., 2014; Chen et al., 2021; Mantovani et al., 2022). Nanodelivery systems are useful because they can provide co-delivery, sequential release, or local triggering so that different pharmacological effects are combined in a controlled way within the same tumor region.

### 3.1 Cytokine- and chemokine-driven regulation

The field initially focused on how individual cytokines induced macrophage phenotypes and later shifted toward cytokine-chemokine networks formed by tumor cells, fibroblasts, endothelial cells, Treg cells, and myeloid cells. The CCL2/CCR2, CSF-1/CSF-1R, and CXCL12/CXCR4 axes mainly influence monocyte recruitment and TAM maintenance (Noy and Pollard, 2014; Cassetta and Pollard, 2018), whereas IFN-γ, IL-4, IL-13, IL-10, and TGF-β further determine TAM functional orientation (Mempel et al., 2024).

This network is strongly time-dependent. Inflammatory signals during early tumor formation may promote antigen presentation and immune surveillance, whereas persistent CSF-1, TGF-β, and metabolic pressure during tumor progression often push TAMs toward immunosuppression, angiogenesis, and matrix remodeling (Mantovani et al., 2017; DeNardo and Ruffell, 2019). Therefore, when nanodelivery systems are used to modulate cytokine networks, release timing and therapeutic window should be considered rather than relying on a single phenotypic measurement.

#### 3.1.1 Immune activation associated with IFN-γ, GM-CSF, and IL-12

IFN-γ is a key cytokine for classical macrophage activation. It upregulates MHC molecules, iNOS, IL-12, and antigen-presentation genes through the JAK/STAT1 pathway, thereby strengthening tumor-antigen processing and T-cell priming. GM-CSF promotes myeloid-cell maturation and antigen presentation, whereas IL-12 connects innate and adaptive immunity by promoting Th1 responses and CD8^+^ T-cell cytotoxicity. These cytokines can cause fever, hypotension, hepatotoxicity, and cytokine-associated inflammation after systemic administration, so local delivery, sustained release, or TAM-targeted delivery has clear pharmacological relevance.

STAT1 signaling driven by IFN-γ is generally associated with enhanced antigen presentation and increased nitric oxide synthase expression. GM-CSF increases myeloid maturation and co-stimulatory molecules, and IL-12 supports Th1 and cytotoxic T-cell responses (Murray et al., 2014; Locati et al., 2020). Because these cytokines are potent but risky when broadly exposed, recent work has favored local delivery, nanoparticle encapsulation, or tumor-microenvironment-responsive release to raise intratumoral concentrations while limiting nonspecific immune activation in the circulation and normal tissues (Irvine and Dane, 2020; Martin et al., 2020).

#### 3.1.2 Suppressive and tissue-repair programs associated with IL-4, IL-13, IL-10, and TGF-β

IL-4 and IL-13 activate STAT6 signaling and induce Arg-1, CD206, and tissue-repair genes. IL-10 and TGF-β maintain immunosuppression through STAT3, SMAD, and related transcriptional networks. Tumor cells, cancer-associated fibroblasts, and Treg cells can all produce these factors and thereby confer tumor-promoting functions on TAMs. Pharmacologically, blockade of these suppressive signals, or nanodelivery of upstream or downstream pathway modulators, can weaken M2-like programs and shift TAMs toward inflammatory phenotypes.

In tumor tissue, IL-4, IL-13, IL-10, and TGF-β do not act in isolation; they cooperate with hypoxia, lactate, adenosine, and matrix stiffness to create a stable tumor-promoting niche (DeNardo and Ruffell, 2019; Mantovani et al., 2022). Blocking a single M2-inducing factor is therefore often insufficient for durable reprogramming. Nanomedicines can combine inhibitory modulators with inflammatory agonists or metabolic regulators so that suppression is reduced and immune stimulation is enhanced within proximate tumor regions.

### 3.2 Pattern-recognition receptor signaling: TLR and STING pathways

Pattern-recognition receptor signaling has expanded TAM reprogramming from the inhibition of tumor-promoting signals to the active induction of antitumor inflammation (Rodell et al., 2018; Shae et al., 2019). TLRs and STING can activate NF-κB, IRF, and type I interferon networks, but they differ in ligand type, intracellular localization, cytosolic delivery requirements, and inflammatory duration; these differences impose distinct requirements on nanocarriers.

Representative studies show that the therapeutic index of innate immune agonists is highly delivery-dependent. R848-loaded nanoparticles increase TAM exposure, whereas endosomolytic STING delivery systems and metal-based immune nanoparticles improve cytosolic delivery or amplify STING signaling (Rodell et al., 2018; Sun et al., 2021; Dosta et al., 2023). Collectively, these studies establish spatially restricted innate immune activation as an important pharmacological principle.

#### 3.2.1 TLR activation and macrophage immune stimulation

TLRs are key receptors for innate recognition of pathogen-associated and damage-associated molecular patterns. TLR7/8 agonists such as R848, TLR9 agonists such as CpG, TLR4 agonists such as MPLA, and TLR3 agonists such as poly I:C activate NF-κB, IRF, and MAPK signaling through MyD88-or TRIF-associated pathways, thereby promoting TNF-α, IL-12, and type I interferon-related molecules. R848-loaded nanoparticles designed by Rodell and colleagues demonstrated that nanodelivery can polarize TAMs toward an immune-stimulatory state and enhance immunotherapeutic efficacy, providing representative evidence for TAM-targeted innate immune activation (Rodell et al., 2018).

The development of TLR agonists has progressed from conventional immune adjuvants to intratumoral myeloid-cell reprogramming. Loading the TLR7/8 agonist R848 into β-cyclodextrin nanoparticles changed its *in vivo* distribution, increased TAM exposure, and enhanced immune-stimulatory TAM activity while reducing systemic toxicity (Rodell et al., 2018). Strategies that mimic microbial signaling also support the feasibility of using innate immune receptors to confer tumor-suppressive functions on macrophages (Feng et al., 2019).

The intensity and duration of TLR signaling determine the therapeutic window. Moderate activation promotes TNF-α, IL-12, and co-stimulatory molecules, whereas excessive or prolonged activation may induce systemic inflammation, immune-cell exhaustion, or tolerance-like states (Irvine and Dane, 2020). Accordingly, the design question for TLR nanomedicines has shifted from whether activation is possible to which cells are activated, at what dose, and for how long.

#### 3.2.2 STING activation and type I interferon-mediated antitumor immunity

The STING pathway detects cytosolic DNA-associated signals and activates the TBK1-IRF3 axis, inducing type I interferons and inflammatory chemokines. It participates in dendritic-cell maturation, antigen cross-presentation, T-cell priming, and TAM remodeling. STING agonists such as cGAMP and cyclic dinucleotides have potent immune-activating potential, but free drugs show poor cellular uptake, inefficient cytosolic delivery, and rapid clearance. STING-NPs enhance cGAMP cytosolic delivery (Shae et al., 2019), cyclic dinucleotide-manganese particles amplify STING signaling through manganese ions (Sun et al., 2021), and ultrasound-microbubble-decorated nucleotide nanocomplexes integrate image guidance with STING activation (Li et al., 2022). Together, these systems demonstrate that nanodelivery can increase effective STING agonist exposure in tumor tissue and immune cells and promote the conversion of immunosuppressed tumors into immune-active lesions.

The STING nanodelivery field has developed a clear trajectory. Early studies used endosomolytic polymersomes to improve cytosolic delivery of cyclic dinucleotides and thereby strengthen STING activation and antitumor immunity (Shae et al., 2019). Subsequent cyclic dinucleotide-manganese particles used manganese ions to amplify cGAS-STING signaling, PEG-lipid nanodiscs emphasized tumor penetration and immune priming, and polymer-drug conjugates improved stability and the therapeutic window after intravenous administration through cleavable linkers (Sun et al., 2021; Dane et al., 2022; Dosta et al., 2023).

These studies indicate that STING agonist efficacy depends not only on the ligand itself but also on whether sufficient exposure is achieved in the cytosol of the appropriate cells. For TAM reprogramming, STING activation can alter myeloid-cell states through type I interferons, CXCL9/CXCL10, and inflammatory cytokines (Li et al., 2022; Yang et al., 2023), thereby supporting T-cell infiltration and antigen presentation.

### 3.3 Kinase and transcription factor pathways

Kinases and transcription factors sit at the convergence of cytokine, metabolic, and pattern-recognition receptor signals and are therefore pharmacologically tractable nodes for TAM state switching. PI3Kγ, STAT3, STAT6, NF-κB, and related pathways influence TAM cytokine expression, antigen presentation, chemokine production, and T-cell-supporting functions (Kaneda et al., 2016; De Henau et al., 2016; Locati et al., 2020).

Compared with single receptor-ligand interactions, interventions at these signaling nodes are more likely to produce systemic effects because the same pathways have basal functions in multiple immune cells and normal tissues. Nanodelivery can reduce systemic exposure by increasing TAM accumulation and enabling microenvironment-responsive release, but pharmacodynamic evaluation must show that the intervention remodels intratumoral myeloid states rather than broadly suppressing peripheral immunity (Martin et al., 2020; Rannikko and Hollmén, 2024).

#### 3.3.1 PI3Kγ, JAK/STAT, and NF-κB signaling

PI3Kγ is a key pharmacological target in immunosuppressive TAMs. Kaneda and colleagues found that PI3Kγ functions as a molecular switch controlling the balance between macrophage immunosuppression and immune stimulation; inhibiting PI3Kγ reduces immunosuppressive mediators, enhances T-cell cytotoxicity, and synergizes with immune checkpoint blockade (Kaneda et al., 2016). The JAK/STAT pathway produces opposite effects depending on the cytokine stimulus: STAT1 is generally associated with IFN-γ-induced inflammation, whereas STAT3 and STAT6 are more often linked to immunosuppression and tissue repair. NF-κB is central to inflammatory activation and TLR responses, but excessive activation can cause systemic inflammation, requiring spatially and temporally controlled delivery.

PI3Kγ research provides a mechanistic foundation for pharmacological TAM reprogramming. Kaneda et al. and De Henau et al. independently showed that inhibiting myeloid PI3Kγ reduces suppressive transcriptional programs and improves checkpoint blockade efficacy, shifting PI3Kγ from a general kinase target to a regulator of TAM immune state (Kaneda et al., 2016; De Henau et al., 2016). CSF-1R blockade can also shift TAMs in pancreatic cancer models from tumor-promoting states toward phenotypes with greater antigen presentation and T-cell support, increasing responses to T-cell checkpoint therapy (Zhu et al., 2014).

JAK/STAT and NF-κB signaling are context-dependent in TAMs. STAT1 and NF-κB often participate in inflammatory activation, whereas STAT3 and STAT6 are more often associated with immunosuppression, tissue repair, and angiogenesis. Excessive or nonselective NF-κB activation may cause systemic inflammation and tissue damage (Locati et al., 2020; Mantovani et al., 2022). These signaling nodes are suitable for combination with nanodelivery because local enrichment and responsive release can lower the risk of systemic pathway perturbation.

#### 3.3.2 HIF-1α, Notch, and MAPK-related regulation

Hypoxia is a major pathological feature of solid tumors and regulates TAM metabolism, angiogenesis, and immunosuppression through HIF-1α and HIF-2α. Notch, MAPK, JNK, and AP-1 pathways also participate in macrophage inflammatory activation, differentiation, and functional maintenance. Compared with single-target inhibition, nanoplatforms can simultaneously deliver multiple modulators or co-deliver pathway inhibitors with immune agonists to generate more stable TAM functional remodeling.

HIF-related signaling connects hypoxia, metabolism, and immunosuppression. Lactate and low oxygen can promote TAM expression of VEGF, Arg-1, and tissue-repair programs (Colegio et al., 2014), while also altering tumor vasculature and metastatic potential (Wenes et al., 2016). TAMs can also reinforce tumor hypoxia and aerobic glycolysis, indicating a positive feedback loop between TAMs and tumor cells that may reduce the efficacy of immunotherapy and drug delivery (Jeong et al., 2019).

Notch, MAPK, JNK, and AP-1 pathways influence TAM inflammatory tone, survival, and cytokine expression according to upstream stimuli and tissue context. Because these pathways also have important roles in normal cells, systemic interference can readily cause off-target effects. Combining these interventions with TAM-targeted nanodelivery, hypoxia-responsive release, or local external triggering is therefore a rational route to improve pharmacological selectivity (van der Meel et al., 2019; Martin et al., 2020).

### 3.4 Metabolic and redox regulation

Metabolic and redox regulation expands TAM research from immune receptor signaling to the level of tumor ecosystem control. Aerobic glycolysis, hypoxia, and lactate accumulation in tumor cells can change TAM transcriptional programs and angiogenic functions, whereas TAMs can in turn influence tumor oxygen metabolism, vascular morphology, and immune-cell entry (Colegio et al., 2014; Wenes et al., 2016; Jeong et al., 2019).

These changes also provide release cues for smart nanodelivery systems. Microenvironmental features such as pH, lactate, hypoxia, ROS, and GSH can be used to construct conditionally responsive carriers (van der Meel et al., 2019; Zhang et al., 2024), but their spatial heterogeneity and treatment-induced dynamics require validation by combining material responsiveness with *in vivo* pharmacokinetic and tissue-imaging analyses (Mahmoudi et al., 2023).

#### 3.4.1 Lactate, hypoxia, and metabolic reprogramming of TAMs

Tumor metabolic states directly shape TAM function. Enhanced glycolysis in tumor cells leads to lactate accumulation, and lactate can promote immunosuppressive and pro-angiogenic TAM programs. Hypoxia, acidic pH, lipid-metabolism remodeling, and amino-acid metabolic abnormalities jointly define TAM metabolic adaptation. Metabolic regulation is important not only for identifying pharmacological targets but also for designing triggerable nanodelivery systems; acidic, hypoxic, and lactate-rich environments can be exploited for selective drug release (Colegio et al., 2014; Qian et al., 2024).

Colegio et al. identified tumor-derived lactic acid as a functional driver of TAM polarization, linking the Warburg effect to immunosuppressive and angiogenic TAM programs (Colegio et al., 2014). Later work expanded this concept by showing that metabolic states differ across TAM subtypes and that lipid metabolism, amino-acid metabolism, and mitochondrial function can influence TAM phenotype and therapeutic response (Qian et al., 2024). Therefore, nanomedicines that target metabolism should not be evaluated only by changes in lactate or pH, but by whether metabolic remodeling translates into functional immune activation.

#### 3.4.2 ROS/GSH balance and redox-sensitive TAM modulation

Redox status is an important bridge between nanodelivery and TAM regulation. Altered ROS and GSH levels in tumor tissue and immune cells influence inflammatory signaling, antigen presentation, and cell death, and they can also trigger carrier disassembly or drug release. ROS-responsive, GSH-responsive, and enzyme-responsive nanosystems can release drugs in specific tumor or intracellular environments, theoretically improving TAM-localized drug exposure and reducing toxicity in normal tissues.

The redox biology of TAMs is dual in nature. Moderate ROS may participate in inflammatory signaling and tumor-cell killing, whereas sustained oxidative stress can promote tissue damage, immunosuppression, and macrophage dysfunction. GSH-rich intracellular environments are frequently used to cleave disulfide bonds and release nucleic acids or small molecules, but redox levels differ across tumor types and immune-cell states (Mahmoudi et al., 2023). As a result, redox-responsive nanomedicines should report release kinetics in biologically relevant conditions and verify whether intracellular release occurs in TAMs rather than in non-target phagocytes.

## 4 Smart nanodelivery systems for TAM-targeted pharmacological intervention

A smart nanodelivery system for TAM-targeted pharmacological intervention should address four linked questions: how the drug remains stable *in vivo*, how it accumulates in tumor tissue, how it preferentially enters or acts on TAMs, and how it releases active cargo at the appropriate time and location. Nanoscale size alone is insufficient to define smart delivery. The design logic shown in Figure 2 emphasizes that true smartness should be reflected in targeting, responsiveness, coordination with pharmacology, and translational feasibility.

**FIGURE 2:**
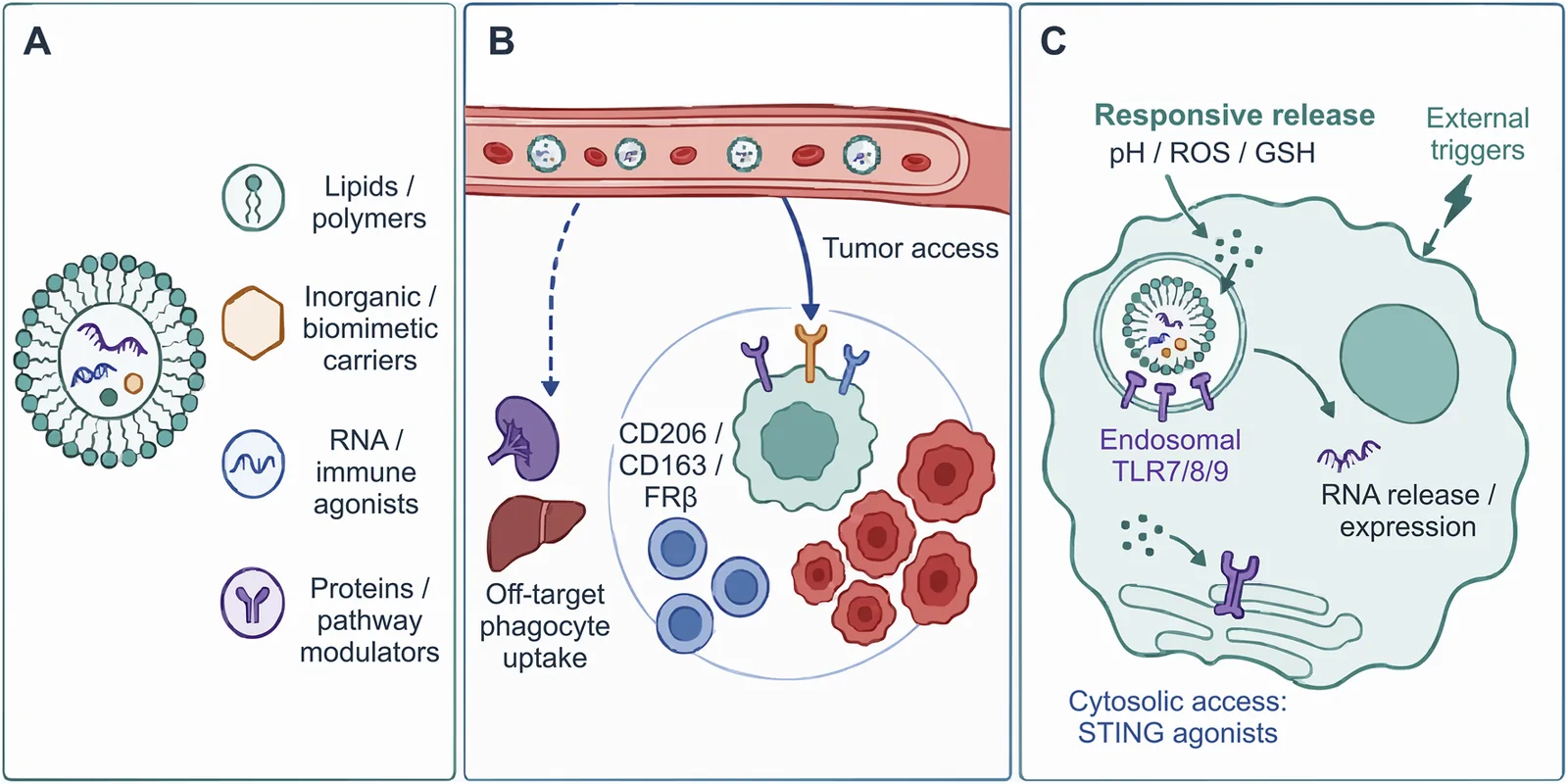
Design steps for TAM-directed nanodelivery. **(A)** Carrier selection is matched to cargo properties. **(B)** Tumor access and receptor-associated enrichment compete with uptake by normal phagocytes; enrichment does not establish cell specificity. **(C)** Local or responsive release and intracellular access determine cargo availability at its site of action. Endosomal TLR7/8/9 signaling is distinguished from cytosolic delivery of STING agonists and RNA. STING is associated with the endoplasmic reticulum. ROS, reactive oxygen species; GSH, glutathione; FRβ, folate receptor β.

The field has moved beyond the assumption that nanoparticles will automatically accumulate in tumors through the EPR effect. Quantitative analyses have shown that only a small fraction of the injected nanoparticle dose reaches tumors in many settings, and the mononuclear phagocyte system remains a major barrier to tumor delivery (Wilhelm et al., 2016). For TAM-targeted therapy, this barrier is also an opportunity because tumor macrophages actively internalize nanomaterials. Effective design must therefore distinguish beneficial intratumoral TAM uptake from off-target capture by liver Kupffer cells, splenic macrophages, and bone marrow phagocytes.

### 4.1 Why nanodelivery is needed for TAM modulation

TAM-modulating agents often have strong immune activity and narrow therapeutic windows. Free TLR or STING agonists may induce systemic inflammation, cytokines may cause dose-limiting toxicity, nucleic acid therapeutics cannot easily cross macrophage membranes, and small-molecule inhibitors may affect myeloid cells in normal tissues. Nanodelivery can change *in vivo* distribution through encapsulation, surface modification, sustained release, and local activation (Nam et al., 2019; Irvine and Dane, 2020), thereby improving pharmacokinetic/pharmacodynamic relationships and enabling potent immunomodulators to act within tumors while reducing systemic exposure.

Nanomedicines also face intrinsic challenges. The EPR effect varies substantially across tumors and patients, nanoparticles are frequently cleared by the hepatic and splenic mononuclear phagocyte system, protein corona formation can change surface identity and cellular uptake, and high interstitial pressure and dense stroma limit penetration in solid tumors. TAM-targeted nanomedicines should therefore combine material design with tumor biology, PK/PD modeling, and patient stratification rather than relying on carrier complexity alone (Shi et al., 2017; van der Meel et al., 2019; Mahmoudi et al., 2023).

### 4.2 Nanocarrier platforms for TAM-targeted delivery

Nanocarrier selection has progressed from simple drug encapsulation to rational matching between cargo properties, release requirements, and target-cell biology. TLR agonists often require controlled local exposure, STING agonists require cytosolic delivery, nucleic acids require protection and endosomal escape, cytokines require activity preservation, and immune-checkpoint proteins or fragments require structural stability. Table 1 compares the major carrier platforms in this context, reinforcing that a suitable TAM-targeted platform should be selected according to the pharmacological action of the cargo rather than solely on the basis of particle size or material category.

**TABLE 1:** Nanocarrier platforms for TAM-targeted delivery. Representative sources are cited in each row; platform-level advantages, limitations, and proposed solutions are literature-based synthesis.

Platform	Suitable cargos	Main advantages	Main limitations
Liposomes (Irvine and Dane, 2020)	Small molecules, proteins, TLR/STING agonists	Good biocompatibility and relatively mature clinical foundation	Stability, leakage, and targeting efficiency require optimization
Lipid nanoparticles (LNPs) (Han et al., 2025; Gong et al., 2021)	mRNA, siRNA, immune adjuvants	Strong nucleic acid delivery capacity and transient expression	Pronounced liver accumulation; TAM selectivity requires additional design
Polymeric nanoparticles (Rodell et al., 2018; Shae et al., 2019)	Small molecules, proteins, nucleic acids	Tunable structure, controllable release, and convenient co-delivery	Degradation products, batch variation, and scale-up need evaluation
Inorganic nanoparticles (Zanganeh et al., 2016; Li et al., 2020)	Photothermal agents, photosensitizers, imaging agents, small molecules	Multifunctional, imageable, and compatible with external triggering	Long-term accumulation and chronic toxicity require attention
Metal-organic frameworks (MOFs) (Xu et al., 2024b)	Small molecules, proteins, gas molecules, multi-drug cargos	High loading capacity, tunable pores, and responsive release	*In vivo* stability and safety require systematic validation
Exosomes/extracellular vesicles (Xu et al., 2020; Qi et al., 2021)	miRNA, mRNA, proteins, small molecules	Natural delivery features and good biocompatibility	Complex sources, low yield, and difficult quality control
Cell-membrane-coated nanoparticles (Fang et al., 2023)	Multiple cargos	Biomimetic targeting, immune evasion, and homologous recognition	Complex preparation and difficult control of membrane source and batch consistency
Hydrogels/local delivery systems (Zúñiga et al., 2022; Nam et al., 2019)	Cytokines, antibodies, nanoparticles	Local sustained release; suitable for postoperative or intratumoral therapy	Limited coverage of systemic metastatic lesions

#### 4.2.1 Lipid-based nanocarriers and lipid nanoparticles

Liposomes and lipid nanoparticles (LNPs) have good biocompatibility and an established foundation for nucleic acid delivery. They can deliver small-molecule immune agonists, siRNA, mRNA, proteins, and combination cargos. Ionizable lipids in LNPs promote endosomal escape in acidic endosomal environments, making them important platforms for mRNA and siRNA delivery. However, LNPs often accumulate in the liver, and tuning their size, lipid composition, surface ligands, and administration route to redirect them toward tumor TAMs remains a major challenge. Recent *in situ* CAR-macrophage therapy using co-delivery of mRNA and immunostimulants (Han et al., 2025) demonstrates the potential of LNPs for programming TAM function *in vivo*.

The history of LNPs in vaccines and RNA therapeutics provides valuable formulation principles for TAM-targeted applications, but cancer immunotherapy imposes additional requirements. A TAM-oriented LNP should not only protect RNA during circulation but also avoid excessive hepatic uptake, penetrate tumor tissue, be internalized by target myeloid cells, and release cargo intracellularly. These requirements have prompted interest in ligand modification, organ-selective lipid chemistry, charge-switchable materials, and local delivery routes (Gong et al., 2021; Irvine and Dane, 2020).

#### 4.2.2 Polymeric, inorganic, and metal-organic framework nanocarriers

Polymeric nanoparticles based on PLGA, PCL, PEGylated polymers, β-cyclodextrin systems, and related materials are structurally tunable, compatible with diverse cargos, and suitable for controlled release. They can deliver TLR agonists, small-molecule inhibitors, proteins, and nucleic acids. Inorganic nanoparticles, including gold nanoparticles, iron oxide nanoparticles, mesoporous silica, and carbon-based materials, are suitable for integrating imaging, photothermal, magnetic, catalytic, or redox functions. Metal-organic frameworks (MOFs) offer high surface area, tunable pore size, and multi-drug loading, and can be designed as pH-, GSH-, or enzyme-responsive systems. Nevertheless, their long-term degradation, safety, and scalable manufacturing require systematic evaluation.

The development of polymeric nanomedicines reflects the transition from carrier stability to immunopharmacological control. For example, β-cyclodextrin nanoparticles can improve R848 distribution and TAM exposure, whereas polymer-STING agonist conjugates use chemical linkers to improve circulation stability and release behavior (Rodell et al., 2018; Dosta et al., 2023). Inorganic and magnetic nanomaterials can also reshape macrophage responses through iron metabolism, ROS generation, or external-field activation, but their long-term retention and chronic inflammatory risk must be carefully assessed (Zanganeh et al., 2016; Mulens-Arias et al., 2021; Hou et al., 2025). Redox-active nanoceria may additionally serve as radioprotective adjuvants by regulating radiation-associated ROS, although their cell-specific and TAM-related effects require further investigation (Alvandi et al., 2024).

#### 4.2.3 Biomimetic nanocarriers and extracellular vesicle-based systems

Biomimetic nanocarriers use cell-membrane coating, exosomes, or extracellular vesicles to mimic natural cell-cell communication and can improve biocompatibility and immune evasion. Coating nanoparticles with macrophage, tumor-cell, red-blood-cell, or platelet membranes can provide inflammatory tropism, homologous targeting, or prolonged circulation. Extracellular vesicles can deliver miRNA, mRNA, proteins, and small-molecule drugs, but source heterogeneity, limited yield, and difficult quality control restrict translation (Xu et al., 2020; Qi et al., 2021; Xu et al., 2023; Fang et al., 2023).

For TAM-targeted therapy, biomimetic systems offer conceptual advantages because they can exploit natural macrophage migration, adhesion, or phagocytic pathways. However, this same biological complexity creates regulatory and manufacturing challenges. Membrane protein composition, vesicle source, purification method, storage stability, and batch-to-batch variation can all affect biodistribution and immune effects. Therefore, biomimetic TAM nanomedicines should be evaluated not only by targeting efficiency but also by whether their biological identity remains stable after scale-up and in circulation. Beyond cell-membrane-coated particles and extracellular vesicles, intact macrophages can also be loaded *ex vivo* with drugs or drug-containing nanoparticles and reinfused as living carriers. Their intrinsic tumor-homing and phagocytic capacities may improve delivery into poorly perfused or hypoxic tumor regions. However, payload toxicity, cell-source variability, phenotypic drift, uncontrolled cargo release, and the need for standardized cell expansion, genetic modification, and product-release testing substantially complicate clinical development (Guo and Qian, 2024).

### 4.3 Active targeting strategies toward TAMs

Active targeting is intended to increase TAM exposure of therapeutic cargos rather than merely adding material complexity. TAM-associated receptors such as CD206, CD163, FRβ, CSF-1R, CCR2, and scavenger receptors have been used for ligand- or antibody-mediated targeting. However, none of these targets is absolutely specific to TAMs. Normal tissue macrophages, dendritic cells, or endothelial cells may also express related receptors. Active targeting should therefore be combined with tumor-responsive release, local administration, or dual-targeting strategies, and Table 2 links representative TAM targets with commonly used ligands, cargos, and practical limitations.

**TABLE 2:** TAM-associated targets and ligands for nanodelivery. Representative sources are cited in each row; platform-level advantages, limitations, and proposed solutions are literature-based synthesis.

Target	Common ligands/strategies	Suitable cargos	Advantages and limitations
CD206 (Kumari and Choi, 2022; Zhang et al., 2019)	Mannose and mannosylated polymers	R848, siRNA, small molecules, imaging agents	Commonly used for M2-like TAM targeting; specificity is limited because normal macrophages and some dendritic cells can also express CD206
CD163 (Cassetta et al., 2019; Mantovani et al., 2022)	Anti-CD163 antibodies, peptides, ligand modification	Nucleic acid therapeutics, small molecules, cytokines	More associated with immunosuppressive TAMs; antibody cost and tissue penetration need consideration
FRβ (Kumari and Choi, 2022)	Folate modification	Small molecules, imaging agents, nucleic acids	Small ligand and easy conjugation; expression varies by tumor type
CSF-1R (Zhu et al., 2014)	Antibodies, inhibitors, ligand-mediated delivery	CSF-1R inhibitors, siRNA, combination drugs	Involved in TAM survival and maintenance; may affect normal tissue macrophages
CCR2 (Nywening et al., 2016)	CCR2 ligands or inhibitors	Small molecules, nucleic acids, recruitment-blocking drugs	Suitable for inhibiting monocyte recruitment; indirect effect on mature TAM reprogramming
Scavenger receptors (Tsoi et al., 2016; Monopoli et al., 2012)	Polyanionic materials, peptides, natural uptake mechanisms	Multiple nanocargos	Strong macrophage uptake; relatively weak cell selectivity
CD47/SIRPα axis (Qu et al., 2022; Son et al., 2022)	Antibody fragments, fusion proteins, siRNA	Phagocytosis checkpoint modulators	Can enhance phagocytosis; systemic blockade may cause hematological toxicity

A key lesson from recent nanomedicine is that receptor binding measured *in vitro* does not necessarily predict *in vivo* targeting. Protein corona formation, vascular permeability, interstitial pressure, and mononuclear phagocyte clearance can all alter nanoparticle access to target receptors (Monopoli et al., 2012; Mahmoudi et al., 2023). Thus, TAM targeting should be validated by cell-resolved biodistribution analysis, such as flow cytometry of tumor immune subsets, imaging-based co-localization, or single-cell methods, rather than by whole-tumor fluorescence signals alone.

#### 4.3.1 CD206-, CD163-, and FRβ-mediated targeting

CD206 is a mannose receptor widely used for targeting M2-like TAMs through mannose-modified nanocarriers. CD163 is associated with immunosuppressive macrophages and can be recognized using antibodies or specific ligands. FRβ is expressed in some activated macrophages and TAMs, and folate modification is attractive because folate is small and easy to conjugate. These strategies can increase TAM uptake, but their target specificity is limited because related receptors are also present in normal macrophages and other immune cells. Therefore, receptor-mediated targeting should be interpreted as enrichment rather than absolute specificity.

The development of CD206, CD163, and FRβ targeting also illustrates the limitation of marker-driven design. CD206 positivity may indicate mannose-receptor expression but does not necessarily define a uniformly immunosuppressive or reprogrammable TAM subset. CD163 and FRβ similarly show tumor-type-dependent and tissue-dependent expression. Future ligand design should therefore integrate receptor expression with functional phenotypes, spatial niches, and treatment response, allowing targeting ligands to be selected according to the TAM subtypes that actually drive resistance in a given tumor.

#### 4.3.2 CSF-1R-, CCR2-, and scavenger receptor-mediated targeting

CSF-1R participates in macrophage survival, differentiation, and TAM maintenance and is one of the most common TAM targets in clinical investigation. CCR2 primarily mediates monocyte recruitment and is suitable for blocking TAM replenishment. Scavenger receptors mediate macrophage uptake of polyanionic molecules and some nanomaterials, making them useful for enhancing macrophage delivery but less selective. Pharmacologically, CSF-1R and CCR2 interventions are more related to reducing recruitment or survival, whereas CD206, CD163, and scavenger-receptor targeting are more related to delivering reprogramming cargos.

Clinical and preclinical studies indicate that blocking TAM recruitment or survival can produce compensatory responses. For instance, CSF-1R inhibition may reduce macrophage numbers in some settings but reshape remaining myeloid cells in others, and CCR2 blockade may be less effective once mature TAM populations are established. Therefore, depletion-oriented strategies are increasingly combined with functional reprogramming, checkpoint blockade, or cytotoxic therapy. Nanodelivery can support this transition by delivering inhibitors, agonists, or nucleic acids to specific myeloid compartments with improved spatial control.

### 4.4 Stimuli-responsive release in the tumor microenvironment

Stimuli-responsive release is a defining feature of smart TAM-targeted nanomedicine. Tumors contain acidic extracellular pH, elevated ROS, high intracellular GSH, overexpressed proteases and MMPs, and hypoxic regions. These features can be used to trigger drug release in tumor tissue, in the endosomal/lysosomal compartment, or in specific cell states. An ideal responsive system should remain stable in blood, be activated in tumor tissue or target cells, and display predictable release kinetics.

The challenge is that tumor stimuli are heterogeneous and dynamic. pH, ROS, GSH, enzyme activity, and oxygen tension vary across tumor types, spatial regions, and treatment stages. A responsive system that works in one model may not release efficiently in another. Therefore, release mechanisms should be evaluated with biologically relevant concentrations and *in vivo* validation, and therapeutic outcomes should be connected to measured intratumoral release rather than assumed from *in vitro* material responsiveness alone.

#### 4.4.1 pH-, ROS-, GSH-, enzyme-, and hypoxia-responsive systems

pH-responsive systems can release cargos in acidic tumor interstitium or endosomes/lysosomes. ROS-responsive systems exploit inflammation and oxidative stress. GSH-responsive systems can cleave disulfide bonds in reducing intracellular environments. Enzyme-responsive systems improve tissue specificity by using protease or MMP activity, and hypoxia-responsive systems are suitable for targeting hypoxic TAM niches and immunosuppressive ecosystems. The strongest designs combine stable systemic circulation with conditional intratumoral activation and controlled intracellular release.

For TAM reprogramming, stimuli-responsive release can synchronize pharmacology with the biology of TAM niches. Hypoxic and lactate-rich regions often contain immunosuppressive TAMs, whereas ROS- and enzyme-rich regions may overlap with inflammatory or matrix-remodeling zones. By linking release mechanisms to these microenvironmental features, nanomedicines can theoretically increase the local concentration of TLR agonists, STING agonists, nucleic acids, or pathway inhibitors in the TAM subpopulations most relevant to immune resistance. This potential must be verified through spatial biodistribution and functional immune readouts.

### 4.5 Externally triggered nanoplatforms

Externally triggered nanoplatforms use light, ultrasound, magnetic fields, or radiation to achieve spatially controlled drug release or local tumor damage. Their value for TAM reprogramming is not limited to tumor-cell killing; external triggers can connect tumor-antigen release, innate immune activation, and TAM remodeling into a coordinated immunological cycle. This design is particularly relevant for poorly inflamed tumors in which endogenous danger signals and antigen presentation are insufficient.

#### 4.5.1 Photothermal, photodynamic, sonodynamic, magnetic, and radiation-responsive platforms

Photothermal and photodynamic therapy can induce tumor-cell death, release tumor antigens, and promote immunogenic cell death. Sonodynamic therapy is more suitable for deeper tumors and, when combined with ultrasound-responsive nanomaterials, may promote immunogenic cell death, TAM phagocytosis and reprogramming, and subsequent T-cell infiltration (Wang et al., 2025). Magnetic systems can support imaging, magnetic hyperthermia, and spatial guidance, whereas radiation-responsive nanosystems can be integrated with standard radiotherapy. For TAM reprogramming, these platforms can be paired with TLR agonists, STING agonists, or cytokines to convert local damage signals into systemic antitumor immunity (Li et al., 2020).

Two examples illustrate complementary mechanisms. Li et al. (2022) used nucleotide nanocomplex-decorated ultrasound microbubbles to couple image-guided delivery with STING activation and antitumor T-cell responses. Wang et al. (2024) combined a sonosensitizer with R848 in G5-CHC-R dendrimeric nanovaccines: ultrasound induced immunogenic tumor-cell damage, while hypoxia-sensitive R848 release promoted TAM phenotypic remodeling. In orthotopic pancreatic and breast cancer models, this combination strengthened local and systemic antitumor immunity. These systems distinguish trigger-assisted intracellular delivery from sonodynamic antigen release coupled to myeloid stimulation; their immune effects need not arise exclusively from TAMs.

External stimulation also introduces practical constraints. Light penetration limits phototherapy in deep lesions, ultrasound parameters must be optimized for safety and efficacy, magnetic guidance depends on field strength and tumor location, and radiotherapy may simultaneously promote immune activation and immunosuppressive myeloid recruitment. Therefore, externally triggered TAM nanomedicines should define their clinical scenario clearly, such as accessible tumors, postoperative beds, image-guided local treatment, or combination with radiotherapy.

## 5 Nanomedicine-mediated TAM reprogramming strategies

Nanomedicine-mediated TAM reprogramming can be categorized by cargo into TLR agonists, STING agonists, kinase and transcriptional modulators, nucleic acid therapeutics, cytokines or immunomodulatory proteins, and macrophage immune-checkpoint modulators. Figure 3 illustrates how these cargos converge on complementary pharmacological nodes, while Table 3 summarizes representative nanomedicine strategies that aim to suppress immunosuppressive TAM programs and enhance antigen presentation, inflammatory activation, phagocytosis, and T-cell-supporting functions.

**FIGURE 3:**
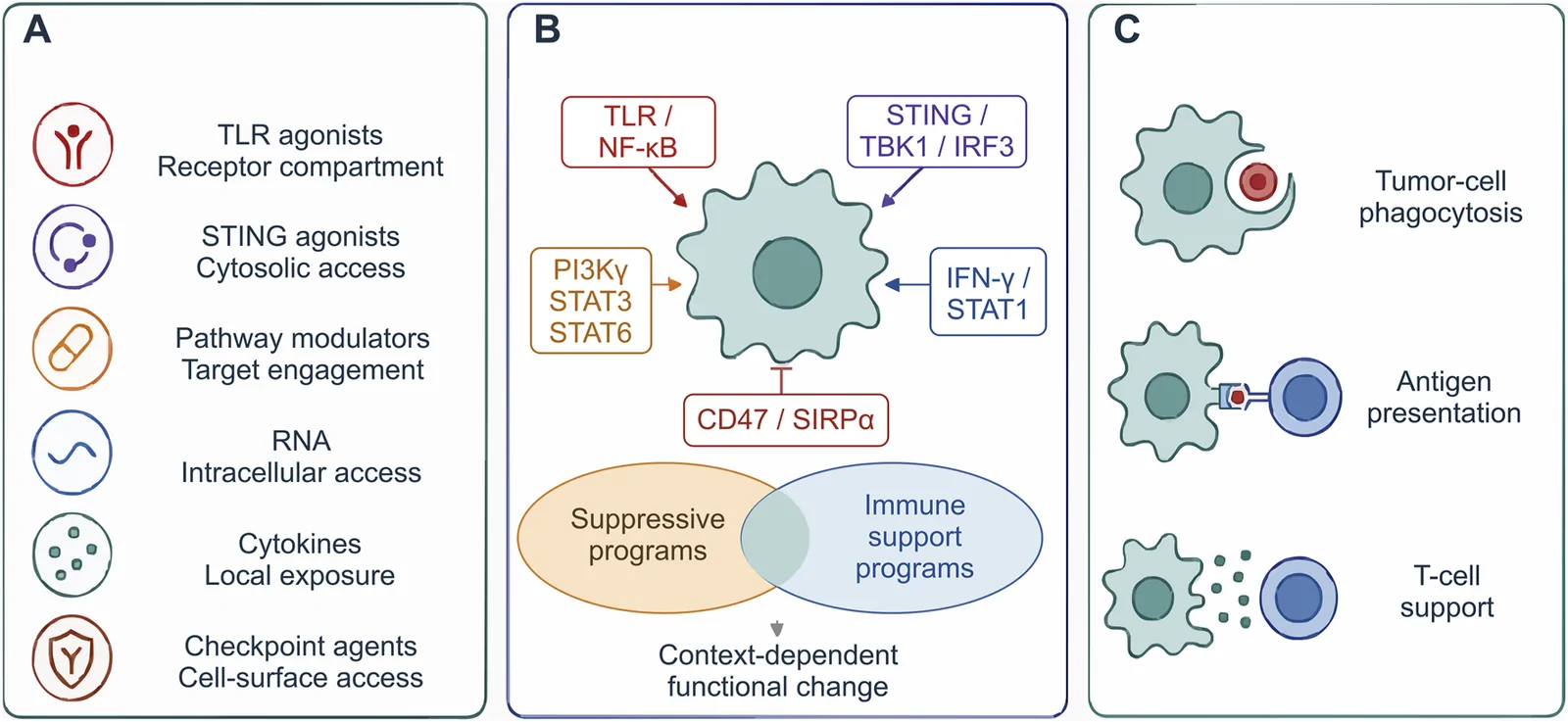
Cargo classes and functional evaluation of TAM-directed interventions. **(A)** Therapeutic cargos have different receptor, intracellular, or cell-surface access requirements. **(B)** Representative pathways influence context-dependent macrophage programs; the schematic does not imply that all pathways act in a single linear sequence. **(C)** Tumor-cell phagocytosis, antigen presentation, and T-cell support are functional endpoints. Changes in activation markers alone do not establish TAM reprogramming. TLR, Toll-like receptor; STING, stimulator of interferon genes.

**TABLE 3:** Representative nanomedicines for TAM reprogramming.

Representative study	Nanoplatform/cargo	Pharmacological mechanism	Immunological outcome	Combination therapy and implication
Rodell et al., 2018	β-Cyclodextrin nanoparticles loaded with the TLR7/8 agonist R848	TLR7/8 activation promotes immune-stimulatory TAM activity	Macrophage IL-12 increased; CD8^+^ T-cell depletion attenuated tumor control	Enhanced immunotherapy responses; effects on other myeloid cells were not excluded
Zhang et al., 2019	Targeted mRNA nanocarriers for macrophage programming	mRNA expression of antitumor functional molecules reshapes macrophage activity *in vivo*	Pro-inflammatory and antitumor functions increased, whereas immunosuppression decreased	Suggests that nucleic acid nanodelivery can achieve *in vivo* TAM functional programming
Kaneda et al., 2016	PI3Kγ pharmacological inhibition (mechanistic non-nano reference)	PI3Kγ acts as a switch between immunosuppression and immune stimulation; macrophage perturbation and transfer support a macrophage contribution	TAM-mediated immunosuppression decreased and T-cell cytotoxicity increased	Provides mechanistic rationale for PI3Kγ inhibitor nanodelivery and ICI combinations
Shae et al., 2019	STING-NPs delivering cGAMP	Improved cytosolic delivery of STING agonists and type I interferon responses	Tumor immunogenicity and innate immune activation increased	Shows potential synergy with immune checkpoint blockade
Sun et al., 2021	Cyclic dinucleotide-manganese particles	Manganese amplifies STING activation and induces local and systemic immunity	T-cell responses and tumor inhibition were promoted	Represents a metal-based immunonanomedicine strategy
Han et al., 2025	LNP co-delivery of CAR mRNA and immunostimulant	*In situ* engineering of TAMs into CAR macrophages with simultaneous immune activation	Phagocytosis and antitumor immunity were enhanced	Suggests that *in vivo* immune-cell engineering may become a new direction for TAM nanotherapy
Xu et al., 2024b	PAZ@Fe-MOF nanoparticles	Improved pazopanib delivery and suppressed M2-associated ATF6–TGFBR1–SMAD3 signaling and CCL17/CCL22/CCL24 expression	Intratumoral M2-like macrophages were reduced and the suppressive immune microenvironment was remodeled	Demonstrates that nanodelivery of a targeted drug can produce indirect TAM-modulating effects beyond improved drug exposure

The development of these strategies shows a gradual increase in mechanistic precision. Early nanomedicines mainly delivered innate immune agonists to broadly activate macrophages. Later systems delivered pathway inhibitors, siRNA, mRNA, or checkpoint modulators to regulate specific molecular programs. The most recent direction is *in situ* immune-cell engineering, in which nanocarriers transiently program macrophages in the tumor to acquire tumor-recognition or enhanced phagocytic functions. This evolution moves TAM nanomedicine from nonspecific immune stimulation toward programmable immunopharmacology.

### 5.1 Delivery of TLR agonists

TLR agonists are among the most mature cargos for TAM reprogramming. R848, a representative TLR7/8 agonist, induces TAM expression of inflammatory mediators and co-stimulatory molecules, promotes inflammatory activation, and supports T-cell activation. Rodell and colleagues loaded R848 into β-cyclodextrin nanoparticles, achieving TAM enrichment and increased macrophage IL-12 expression in multiple tumor models and enhancing immunotherapy responses (Rodell et al., 2018). CD8^+^ T-cell depletion attenuated tumor control, supporting a link between macrophage modulation and adaptive immunity. However, the findings do not exclude effects on other myeloid cells, including dendritic cells.

Other TLR agonists, including CpG, MPLA, and poly I:C, can also be delivered by nanocarriers. CpG activates dendritic cells and macrophages through TLR9, MPLA signals through TLR4 as a relatively moderate immune adjuvant, and poly I:C induces type I interferon through TLR3-and MDA5-like pathways. Future designs should compare how different TLR combinations affect TAMs, dendritic cells, and T cells, while avoiding excessive inflammation or immune tolerance.

A key pharmacological issue is that TLR agonists can activate multiple immune-cell types and may produce opposing outcomes depending on context. Localized TAM exposure can promote antitumor inflammation, but broad systemic activation can lead to cytokine release and toxicity. Therefore, TLR nanomedicines should report not only tumor growth inhibition but also cytokine kinetics, liver and spleen inflammation, and the persistence of immune activation after dosing. TAM-associated activity therefore needs to be distinguished from broader immune activation by comparing cargo uptake and functional responses in macrophages, monocytes, dendritic cells, and T cells.

### 5.2 Delivery of STING agonists

STING agonists activate type I interferon signaling and increase tumor immunogenicity. Free cyclic dinucleotide drugs have difficulty crossing cell membranes and reaching cytosolic STING, making them highly dependent on delivery systems. STING-NPs enhance cGAMP cytosolic delivery, cyclic dinucleotide-manganese particles amplify STING signaling through manganese ions, and ultrasound microbubble-decorated nucleotide nanocomplexes integrate image guidance with STING activation (Shae et al., 2019; Sun et al., 2021; Li et al., 2022).

In the context of TAM reprogramming, STING activation is valuable not only because it stimulates dendritic cells but also because inflammatory cytokines, chemokines, and type I interferon networks can reshape myeloid-cell states. When combined with anti-PD-1 or anti-PD-L1 therapy, STING nanomedicines may increase antigen presentation, T-cell infiltration, and inflammatory activation, converting cold tumors into hotter lesions that are more responsive to checkpoint blockade.

The recent development of STING nanomedicines emphasizes the importance of formulation chemistry. Endosomolytic polymersomes, lipid nanodiscs, metal-coordination particles, and polymer-drug conjugates each address a different barrier, including cytosolic access, tumor penetration, immune amplification, or intravenous stability (Shae et al., 2019; Sun et al., 2021; Dane et al., 2022; Dosta et al., 2023; Chen et al., 2024). For clinical translation, the optimal strategy will likely depend on tumor location, dosing route, safety window, and whether local or systemic antitumor immunity is the therapeutic goal.

### 5.3 Delivery of kinase and transcriptional modulators

Modulators of PI3Kγ, STAT3, STAT6, CSF-1R, HIF-1α, and NF-κB can directly change TAM signaling networks. PI3Kγ inhibitors can relieve TAM-mediated immunosuppression and enhance T-cell cytotoxicity. STAT3 inhibition or silencing may reduce IL-10, VEGF, and other tumor-promoting factors. CSF-1R inhibition can reduce TAM survival or remodel TAM composition, and HIF-1α modulation is suitable for intervention in hypoxic TAM niches (Kaneda et al., 2016; De Henau et al., 2016). In the non-nanoparticle mechanistic study by Kaneda et al. (2016), macrophage perturbation and transfer experiments supported a macrophage contribution to T-cell activation. De Henau et al. (2016) demonstrated a broader suppressive-myeloid mechanism. These findings support the rationale for pathway modulation but do not by themselves establish TAM-selective delivery by a nanocarrier.

The shared problem of these pathway modulators is broad systemic activity that may affect normal myeloid cells and tissue homeostasis. Nanodelivery can reduce off-target risk by TAM targeting, local release, and low-dose combinations. A particularly promising direction is co-delivery of pathway inhibitors with TLR or STING agonists so that relief of immunosuppression and active immune stimulation occur simultaneously within the same tumor region.

This strategy should be evaluated with attention to pathway specificity and immune balance. For example, inhibiting STAT3 may reduce immunosuppressive cytokines but could also affect dendritic cells or T cells depending on dose and exposure. CSF-1R blockade may reduce TAM abundance but may also cause compensatory recruitment of other myeloid cells. Thus, nanomedicines that deliver pathway modulators should report cell-type-specific target engagement and functional immune outcomes rather than relying only on bulk tumor markers. Changes in recruitment or survival should also be distinguished from functional reprogramming of surviving TAMs.

### 5.4 Delivery of nucleic acid therapeutics

Nucleic acid therapeutics provide a way to regulate TAM-associated genes with high specificity. siRNA, miRNA, antisense oligonucleotides, mRNA, and gene-editing systems can silence suppressive pathways, restore inflammatory programs, or transiently express antitumor proteins. Because nucleic acids are vulnerable to nuclease degradation, have negative charge, and require intracellular or cytosolic access, nanocarriers are central to their *in vivo* application.

#### 5.4.1 siRNA, miRNA, and antisense oligonucleotides

siRNA or antisense oligonucleotides can silence STAT3, CD47, CSF-1R, CCR2, HIF-1α, or other immunosuppression-related genes. miRNAs can shift macrophage polarization through network-level regulation. These approaches are attractive because they can address targets that are difficult to inhibit with small molecules or antibodies. However, successful TAM modulation requires protection during circulation, selective delivery to tumor myeloid cells, endosomal escape, and sufficient duration of gene silencing.

Recent designs increasingly combine targeting ligands with pH-, GSH-, or enzyme-responsive release to enhance intracellular delivery in macrophages. Nevertheless, nucleic acid delivery to TAMs must be distinguished from delivery to liver Kupffer cells and splenic macrophages. Cell-resolved biodistribution, target mRNA/protein knockdown in sorted TAMs, and functional evidence of improved antigen presentation or T-cell activation are required to establish a convincing mechanism.

#### 5.4.2 mRNA and gene-editing strategies

mRNA delivery enables TAMs to transiently express antitumor proteins or transcriptional regulators without genomic integration, making it reversible and adjustable. Zhang and colleagues reported targeted mRNA nanocarriers that genetically programmed macrophages to express antitumor functional molecules, providing early evidence for *in vivo* TAM programming (Zhang et al., 2019). More recently, LNP co-delivery of CAR mRNA and an immunostimulant was proposed to generate CAR macrophages *in situ* within tumors, suggesting that *in vivo* immune-cell engineering may become a new direction for TAM-targeted nanomedicine (Han et al., 2025). Gene-editing systems such as CRISPR/Cas offer long-term regulatory potential, but their delivery efficiency, safety, and off-target effects require cautious evaluation.

For mRNA and gene-editing strategies, reversibility is a major design consideration. Transient mRNA expression may be safer for immune activation than permanent editing, especially when the target cell population includes heterogeneous macrophage subsets. Conversely, gene editing may be useful for durable removal of suppressive checkpoints or metabolic programs. Comparisons of transient and durable approaches require matched pharmacodynamic endpoints, including phagocytosis, antigen presentation, cytokine release, and T-cell response.

### 5.5 Delivery of cytokines and immunomodulatory proteins

IFN-γ, IL-12, and GM-CSF can markedly enhance macrophage inflammatory activation, antigen presentation, and T-cell support. However, systemic cytokine exposure often causes serious toxicity, and these proteins have short half-lives and poor tissue selectivity. Nanoplatforms can increase local tumor concentrations through encapsulation, fusion-protein anchoring, hydrogel-based sustained release, or TAM-targeted delivery. Compared with small molecules, proteins and cytokines are more sensitive to formulation conditions, so design should address protein conformation, shear stress during encapsulation, and long-term storage stability.

IL-15 provides a complementary route through activation of NK cells and CD8^+^ T cells. Liu et al. (2018) delivered an IL-15 plasmid using DOTAP/MPEG-PLA nanoparticles in CT26 colon cancer models, increasing lymphocyte activity and intratumoral IFN-γ, TNF-α, and IL-12 alongside tumor control. These findings support immune-microenvironment remodeling, but do not establish direct TAM reprogramming. Increased lymphocyte-derived IFN-γ could indirectly reinforce macrophage antigen presentation and inflammatory activity, providing a rationale for combining IL-15 delivery with TAM-directed TLR/STING agonists or suppressive-pathway inhibitors. Such combinations require macrophage-resolved functional assays to establish the contribution of TAMs (Liu et al., 2018; Locati et al., 2020).

Cytokine delivery has gradually shifted from high-dose systemic administration to localized immune programming. For TAM-targeted therapy, the aim is not simply to raise cytokine levels but to coordinate cytokine exposure with antigen release, myeloid-cell activation, and T-cell priming. Therefore, cytokine nanomedicines should be tested in combination with immune checkpoint blockade, radiotherapy, photodynamic therapy, or cancer vaccines under schedules that reflect the sequence of immune activation.

### 5.6 Targeting macrophage immune checkpoints

Macrophage immune checkpoints extend TAM-targeted therapy beyond polarization markers. The CD47/SIRPα axis, CD24/Siglec-10, PD-1/PD-L1, VISTA, MerTK, and LILRB-related pathways regulate tumor-cell engulfment, apoptotic-cell clearance, and immune suppression through distinct mechanisms. Nanodelivery may help localize checkpoint modulation to tumors, reduce systemic toxicity, and enable combinations with inflammatory agonists or T-cell checkpoint inhibitors.

#### 5.6.1 CD47/SIRPα axis and macrophage-mediated phagocytosis

The CD47/SIRPα axis is the canonical macrophage checkpoint. Tumor cells frequently overexpress CD47, which binds SIRPα on macrophages and suppresses phagocytosis. Blocking this axis can restore macrophage-mediated engulfment of tumor cells and may promote antigen presentation and T-cell responses (Qu et al., 2022; Son et al., 2022). Nanodelivery can be used for local delivery of anti-CD47 antibody fragments, SIRPα fusion proteins, or related siRNA, potentially reducing anemia and hematological toxicity associated with systemic CD47 blockade.

Additional myeloid checkpoints, including MerTK, LILRB, Siglec-10/CD24, PD-1/PD-L1, and VISTA, are increasingly considered therapeutic targets. TAM reprogramming should therefore not be limited to increased CD86 or reduced CD206. It should also include restoration of phagocytosis, enhanced antigen processing, and improved T-cell cross-priming. As phagocytosis checkpoint research advances, nanomedicine may become a useful tool for local, multi-target, and lower-toxicity macrophage checkpoint modulation (Xu et al., 2023; Veillette et al., 2026).

The development of macrophage checkpoint therapy also changes the criteria for evaluating TAM-targeted nanomedicine. If the target is CD47/SIRPα or CD24/Siglec-10, then the most relevant pharmacodynamic endpoint is not only polarization marker expression but also engulfment of tumor cells, antigen transfer, dendritic-cell activation, and the emergence of tumor-specific T-cell responses. This requirement favors experimental designs that integrate phagocytosis assays, flow cytometry, imaging, and T-cell functional analysis.

#### 5.6.2 CD24/Siglec-10

Tumor-cell CD24 engages Siglec-10 on macrophages to restrain engulfment. Blocking this interaction increased macrophage phagocytosis of human cancer cells, providing a rationale for localized checkpoint inhibition (Barkal et al., 2019). Nanodelivery should preserve blocking activity and assess tumor-cell engulfment separately from uptake of apoptotic debris.

#### 5.6.3 MerTK

MerTK promotes efferocytosis of apoptotic cells and can thereby support immune tolerance. Zhou et al. (2020) showed that MerTK blockade enhanced tumor-derived cGAMP transfer and P2X7R-dependent STING activation in macrophages, strengthening T-cell responses and anti-PD-1/PD-L1 activity. This mechanism differs from releasing a brake on viable tumor-cell phagocytosis; local delivery would need to balance antitumor inflammation against impaired clearance of dying cells.

#### 5.6.4 VISTA

VISTA can suppress T-cell activity through pH-dependent engagement of PSGL-1, linking checkpoint function to acidic tumor niches (Johnston et al., 2019). Tumor-activated blockade is therefore mechanistically attractive, but efficacy should be assessed through myeloid–T-cell interactions rather than assumed to reflect direct macrophage reprogramming.

#### 5.6.5 LILRB

LILRB family members have distinct ligands and functions. LILRB2 antagonism shifted human tumor-associated myeloid cells toward inflammatory programs and reduced their suppressive activity (Chen et al., 2018). This supports LILRB2-directed delivery as a candidate strategy, while receptor differences between humans and mice require careful model selection.

#### 5.6.6 PD-1/PD-L1 in TAMs

PD-1 expressed by TAMs can inhibit tumor-cell phagocytosis; blockade restored macrophage-dependent antitumor activity in experimental models (Gordon et al., 2017). TAM-associated PD-L1 can instead restrain PD-1-positive T cells. These cellular roles should be distinguished when designing local checkpoint delivery and interpreting combinations with T-cell-directed immunotherapy.

### 5.7 Nanovaccines and indirect TAM remodeling

Not all nanoimmunotherapies are designed to target TAMs directly. Nanovaccines improve antigen stability, lymphatic transport, antigen-presenting-cell uptake, and the co-delivery of tumor antigens and immune adjuvants. Biomimetic nanovaccines and lipid-nanoparticle-based mRNA vaccines can enhance type I interferon signaling, Th1 polarization, and tumor-specific CD8^+^ T-cell responses. These immune changes may indirectly shift TAMs toward inflammatory and antigen-presenting states through increased antigen availability, inflammatory cytokines, and T-cell-derived IFN-γ. Conversely, prolonged vaccine-associated inflammation may also recruit suppressive myeloid cells or activate compensatory immune checkpoints. TAM abundance, functional state, phagocytosis, and spatial interaction with effector T cells should therefore be included as pharmacodynamic endpoints in nanovaccine studies (Xu et al., 2023; Bhattacharya et al., 2026).

Other multifunctional nanosystems can also regulate TAMs indirectly. Ultrasound-responsive nanomaterials may induce immunogenic cell death, enhance macrophage phagocytosis, and remodel the suppressive tumor microenvironment. Metal-organic-framework delivery of pazopanib has been shown to reduce intratumoral M2-like macrophages and suppress associated signaling pathways. Redox-active nanoceria may modify radiation-associated oxidative stress and tissue injury, although direct TAM-specific effects require further validation. These examples highlight the distinction between direct TAM-targeted delivery and indirect TAM remodeling through antigen release, redox regulation, metabolic changes, and broader immune activation (Xu et al., 2024; Wang et al., 2025; Alvandi et al., 2024).

## 6 Pharmacodynamic consequences of TAM reprogramming in cancer immunotherapy

The value of TAM reprogramming is ultimately expressed through pharmacodynamic outcomes. Figure 4 follows this pharmacodynamic sequence from TAM reprogramming to immune-microenvironment remodeling, emphasizing that studies should demonstrate relief of tumor immunosuppression, enhanced antigen presentation, restored CD8^+^ T-cell function, cold-to-hot conversion, and improved efficacy of immune checkpoint blockade or other immunotherapies.

**FIGURE 4:**
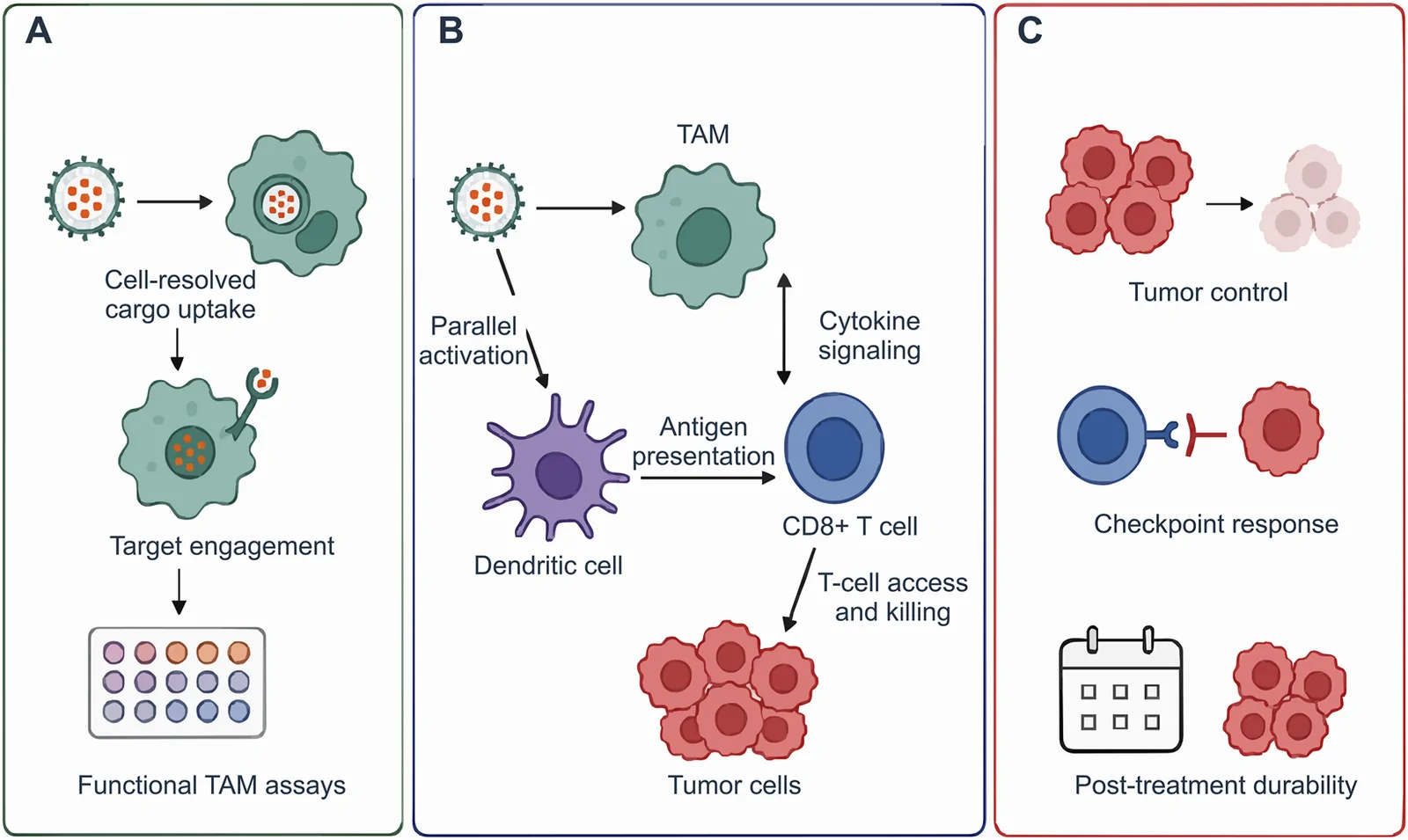
Evidence linking TAM-directed delivery to antitumor outcomes. **(A)** Cell-resolved cargo exposure and target engagement precede functional TAM evaluation. **(B)** Macrophage activity interacts with dendritic cells, lymphocytes, and tumor cells, while parallel immune activation may contribute independently. **(C)** Tumor control, checkpoint response, and durability are downstream outcomes. The illustrated links require experimental validation; tumor response alone does not prove a TAM-specific mechanism. CD8^+^ T cell, cytotoxic T-lymphocyte population.

Following the functional criteria in Section 2.2, a convincing TAM reprogramming study should connect intratumoral drug exposure to TAM state changes, connect TAM changes to T-cell infiltration and function, and connect these immune changes to tumor control, survival, metastasis suppression, or recurrence prevention. Section 7 addresses the corresponding PK/PD considerations.

### 6.1 Remodeling the immunosuppressive tumor microenvironment

After immunosuppressive TAMs are reduced or reprogrammed, inhibitory mediators such as IL-10, TGF-β, Arg-1, VEGF, and PGE2 may decrease. Treg and MDSC recruitment may be reduced, and vascular and stromal barriers may be partially relieved. These changes can decrease T-cell exhaustion and exclusion and improve the baseline environment for immunotherapy. Evaluation should therefore include cytokine profiles, myeloid-cell composition, stromal density, and vascular status rather than relying on a single TAM marker.

TAM reprogramming can also affect the spatial organization of the TIME. A reduction in suppressive macrophages near tumor nests or invasive margins may permit CD8^+^ T cells to contact tumor cells, whereas changes in angiogenic TAMs may normalize vasculature and improve immune-cell trafficking. Spatial transcriptomics and multiplex imaging can therefore provide pharmacodynamic information that bulk flow cytometry or qPCR may miss.

### 6.2 Enhancing antigen presentation and innate immune activation

Immune-stimulatory TAMs can upregulate MHC-I, MHC-II, CD80, CD86, and IL-12, enhancing antigen processing and presentation. Although dendritic cells remain the central cells for T-cell priming, TAM antigen presentation and inflammatory amplification can support local T-cell reactivation. TLR and STING nanomedicines are particularly relevant because they can affect TAMs, dendritic cells, and innate recognition of released tumor antigens at the same time.

The macrophage-resolved assays described in Section 8.3 can test whether enhanced antigen presentation contributes to tumor-specific T-cell responses and durable immune memory.

### 6.3 Promoting CD8^+^ T-cell infiltration and cytotoxicity

Increased CD8^+^ T-cell infiltration is one of the most important pharmacodynamic indicators after TAM reprogramming. Effective T-cell responses are typically reflected by higher CD8^+^ T-cell abundance, increased granzyme B, perforin, and IFN-γ expression, and reduced or functionally reversed exhaustion markers. For combinations with immune checkpoint blockade, studies should further assess T-cell clonal expansion, tumor-antigen-specific responses, and long-term immune memory.

The relationship between TAMs and CD8^+^ T cells is both quantitative and spatial. Increased CD8^+^ T-cell numbers may not translate into cytotoxicity if T cells remain trapped in stromal regions or separated from tumor cells. Therefore, evaluating CD8^+^ T-cell infiltration should include spatial proximity to tumor nests, contact with antigen-presenting myeloid cells, and expression of cytotoxic molecules within tumor-cell-rich areas. These analyses can determine whether TAM reprogramming actually reverses immune exclusion.

### 6.4 Converting cold tumors into hot tumors

Cold tumors typically exhibit low T-cell infiltration, weak antigen presentation, abundant immunosuppressive cells, and poor responsiveness to checkpoint therapy. Hot tumors show stronger inflammatory signaling, antigen presentation, and T-cell infiltration. TAM reprogramming can promote cold-to-hot conversion by relieving myeloid immunosuppression, enhancing innate immune sensing, and improving T-cell entry into tumors. This concept explains why TAM-targeted nanomedicine may enhance anti-PD-1 or anti-PD-L1 therapy.

Cold-to-hot conversion should be considered a multi-dimensional pharmacodynamic phenotype. It may include increased CXCL9/CXCL10, type I interferon signaling, dendritic-cell activation, TAM inflammatory activation, CD8^+^ T-cell infiltration, and tumor-cell killing. Because any single marker can be misleading, integrated immune scoring and spatial pathology are more informative than isolated measurements.

For example, CDNP-R848 enhanced anti-PD-1 activity in otherwise resistant B16.F10 melanoma, consistent with relief of myeloid suppression and improved responsiveness to checkpoint blockade (Rodell et al., 2018). The G5-CHC-R sono-nanovaccine discussed in Section 4.5 combined TAM phenotypic remodeling with antigen release and systemic adaptive immunity in pancreatic and breast cancer models (Wang et al., 2024). Together, these examples support immune sensitization as a practical outcome of cold-to-hot remodeling; neither tumor shrinkage nor altered TAM markers alone establishes a complete spatial or functional conversion.

### 6.5 Synergy with immune checkpoint blockade

Immune checkpoint blockade releases inhibited T cells, but it requires the presence of reactivatable T cells and an immune environment that supports their function. TAM reprogramming can provide this foundation by reducing suppressive mediators, enhancing antigen presentation, promoting T-cell entry, and increasing cytotoxicity. Therefore, combining TAM-targeted nanomedicine with anti-PD-1, anti-PD-L1, or anti-CTLA-4 therapy has clear mechanistic rationale. The timing of these combinations is discussed in Section 7.4.

Combination timing is not a technical detail but a pharmacological determinant of efficacy. If TAM reprogramming is given too early, the inflammatory window may close before T-cell checkpoint blockade is applied. If given too late, immunosuppressive myeloid niches may already have restricted T-cell access. PK/PD modeling can help identify the temporal relationship among nanomedicine exposure, TAM state change, T-cell infiltration, and checkpoint inhibitor efficacy. An additional challenge is the durability of TAM reprogramming. TAM states remain plastic after treatment, and inflammatory activation may induce counter-regulatory pathways, including IDO1–kynurenine metabolism, PD-L1, IL-10, TGF-β, and adenosine signaling, while persistent hypoxia, lactate, CSF-1, and stromal cues can drive repolarization toward tumor-supporting states. A single pulse of an immune-stimulatory reprogramming agent may therefore produce only transient benefit. Future systems should consider sequential or feedback-responsive release, for example by first relieving suppressive metabolic or checkpoint pathways and then delivering TLR or STING agonists, with longitudinal monitoring of TAM states and T-cell function. Durable efficacy should be evaluated after treatment withdrawal and, where appropriate, tumor rechallenge rather than inferred from early marker changes alone (Munn and Mellor, 2016; Cassetta and Pollard, 2023; Chen et al., 2024).

## 7 Pharmacokinetic, biodistribution, and safety considerations

For TAM-targeted nanomedicine, efficacy is determined not only by cargo potency but also by nanoparticle exposure in blood, tumor, liver, spleen, bone marrow, and different immune-cell subsets. A successful formulation must produce sufficient exposure in tumor TAMs while avoiding excessive activation or accumulation in normal myeloid compartments.

The central translational challenge is the mismatch between *in vitro* targeting and *in vivo* distribution. Nanoparticles that bind TAM-associated receptors in culture may be cleared by liver and spleen before reaching tumors. Conversely, particles designed to avoid macrophage uptake may fail to enter TAMs after tumor accumulation. The challenges listed in Table 4 underscore why TAM-targeted nanomedicine requires a balanced design that controls circulation, tissue penetration, myeloid-cell uptake, intracellular release, and systemic immune safety.

**TABLE 4:** Pharmacokinetic, safety, and translational challenges of TAM-targeted nanomedicine. Representative sources are cited in each row; platform-level advantages, limitations, and proposed solutions are literature-based synthesis.

Challenge	Specific manifestation	Impact	Potential solutions
TAM heterogeneity (Cassetta et al., 2019; Ma et al., 2022)	TAM subtypes differ by cancer type, patient, and spatial location	Single-target efficacy can be unstable	Use single-cell and spatial omics to identify functional subtypes and stratify patients
Oversimplified M1/M2 model (Murray et al., 2014; Locati et al., 2020)	CD86/CD206 do not fully represent function	Pharmacodynamic judgment can be incomplete	Combine phagocytosis, antigen presentation, and T-cell activation readouts
Liver and spleen clearance (Tsoi et al., 2016; Wilhelm et al., 2016)	Nanoparticles are heavily taken up by the mononuclear phagocyte system	Tumor delivery efficiency decreases and off-target risk increases	Optimize particle size/surface, use biomimetic coating, local delivery, or activatable targeting
Insufficient target specificity (Mantovani et al., 2022; Kumari and Choi, 2022)	CD206, CD163, and related receptors are also expressed by normal macrophages	Immune perturbation may occur in normal tissues	Use dual targeting, microenvironment-responsive release, and conditional activation strategies
Immunotoxicity (Shae et al., 2019; Son et al., 2022)	TLR/STING agonists may trigger inflammation and cytokine release	Dose-limiting toxicity can occur	Use low-dose combinations, local release, and PK/PD-guided dosing
Insufficient tumor penetration (van der Meel et al., 2019; Martin et al., 2020)	Dense stroma and high interstitial pressure limit nanoparticle diffusion	Deep TAM niches may not be covered	Use size-transformable systems, stromal modulation, and external-trigger assistance
Manufacturing and quality control (Joyce et al., 2024)	Particle size, PDI, loading, release profile, and endotoxin control are difficult	Batch variation affects efficacy and regulatory acceptance	Develop GMP-compatible processes, standardized characterization, and stability studies
Combination timing (Nam et al., 2019; Lee et al., 2025)	The optimal sequence of TAM reprogramming and ICI dosing is unclear	Synergy can be inconsistent	Build PK/PD models and evaluate immune therapeutic windows
Patient selection (Cassetta et al., 2019; Rannikko and Hollmén, 2024)	TAM infiltration and target expression vary among patients	Clinical trial response rates may be limited	Use companion diagnostics, imaging biomarkers, and spatial pathology for selection

### 7.1 Pharmacokinetic challenges of TAM-targeted nanomedicine

Nanomedicine pharmacokinetics are affected by particle size, surface charge, shape, flexibility, PEG density, protein corona, ligand density, and cargo-loading method. An ideal TAM-targeted system should remain stable in blood and avoid premature release, enter tumor tissue and either penetrate the tissue or be taken up by target TAMs, and then achieve endosomal escape or lysosomal release after cellular internalization. If circulation is too short, tumor exposure is insufficient; if circulation is too long, off-target accumulation and long-term toxicity may increase.

PK evaluation should include both carrier and cargo. A carrier may remain in circulation while cargo is released prematurely, or cargo may remain encapsulated but fail to reach the intracellular compartment required for activity. Therefore, dual-label tracking, cargo-release assays, and cell-specific exposure analysis are needed to understand the relationship between nanoparticle disposition and TAM pharmacodynamics.

### 7.2 Biodistribution and off-target accumulation

Liver Kupffer cells and splenic macrophages are major sites of nanoparticle clearance, creating the paradox that a formulation intended to target tumor macrophages may first be captured by normal phagocytes. TAM-associated targets such as CD206, CD163, and scavenger receptors are also expressed by normal macrophages, increasing off-target risk. Strategies to improve tumor selectivity include local administration, dual targeting, tumor-microenvironment-responsive release, cleavable shielding layers, size-transformable systems, and image-guided delivery.

Biodistribution studies should be performed at the level of organs, tissues, and cells. Whole-organ fluorescence or radiolabel signals cannot distinguish tumor-cell uptake from TAM uptake or normal macrophage clearance. Flow cytometry of dissociated tissues, imaging-based co-localization, and analysis of sorted immune-cell subsets are needed to determine whether the formulation achieves meaningful TAM exposure.

### 7.3 Immunotoxicity and inflammatory risk

The safety challenge of TAM reprogramming is specific: efficacy depends on immune activation, but excessive activation can cause systemic inflammation, cytokine release, hepatosplenic inflammatory injury, and autoimmune-like responses. TLR and STING agonists require especially careful control of local exposure and release rate. CD47/SIRPα blockade may affect clearance of red blood cells and platelets. Inorganic or poorly degradable materials require evaluation of long-term accumulation and chronic toxicity. Safety assessment should include hematology, liver and kidney function, inflammatory cytokines, histopathology, complement activation, and long-term immune-memory or autoimmunity risk.

Immunotoxicity also depends on schedule and combination context. A dose that is safe as monotherapy may become inflammatory when combined with radiotherapy, chemotherapy, or checkpoint blockade. Nanomedicine studies should therefore assess cytokine kinetics, repeated-dose tolerance, organ inflammation, and recovery after immune activation. Reporting only body weight and major-organ histology is insufficient for immunologically active formulations.

### 7.4 Dose, schedule, and combination timing

Dose and timing are central pharmacological questions in combination therapy. Too low a dose of a TAM reprogramming agent may fail to alter the immune microenvironment, whereas too high a dose may induce inflammatory toxicity. Administering the TAM reprogramming agent before ICI may create a better T-cell environment, simultaneous administration may produce immediate synergy, and delayed administration may be appropriate after radiotherapy, chemotherapy, or photodynamic therapy releases tumor antigens. PK/PD models should integrate nanomedicine exposure, TAM state changes, T-cell infiltration, and tumor-volume dynamics into a unified predictive framework.

Dose optimization should also consider spatial heterogeneity. Tumors with high stromal density or poor vascularization may require local administration or external triggering, whereas highly vascularized tumors may respond to systemic nanocarriers. Repeated dosing may be required to maintain TAM reprogramming, but repeated innate immune activation can induce tolerance or toxicity. These considerations underscore the need to define an immune therapeutic window rather than simply maximizing nanoparticle dose.

Published regimens illustrate why exposure duration and treatment order should be evaluated separately. Rodell et al. (2018) examined single and repeated CDNP-R848 dosing and demonstrated benefit with anti-PD-1, but did not establish an optimal before-versus-after ICI sequence. As a local sustained-release comparator, the hydrogel-based TransCon TLR7/8 agonist maintained resiquimod exposure over several weeks after one intratumoral dose and enhanced systemic immunotherapy in mouse models (Zúñiga et al., 2022). This is a microsphere depot rather than a TAM-selective nanoparticle. A direct sequencing comparison by Lee et al. (2025) tested anti-PD-L1 at 0, 1, 3, 5, or 7 days after 10 Gy irradiation in CT26 and 4T1 models; the day-5 schedule, coinciding with peak PD-L1 expression, produced the strongest therapeutic interaction. That non-nanomedicine study provides a timing principle rather than a validated schedule for TAM nanotherapy. Comparisons of pretreatment, concurrent treatment, and delayed treatment at matched cumulative doses remain necessary for each TAM-directed formulation.

## 8 Preclinical models and evaluation methods

Preclinical evaluation should cover delivery efficiency, TAM reprogramming, systemic immune response, and antitumor efficacy. Only when a nanoplatform reaches target cells and produces functional immune changes can it be considered a credible immunotherapy sensitization strategy.

The choice of model should match the biological question. *In vitro* systems are useful for studying uptake, release, and immediate signaling, whereas *in vivo* tumor models are required for evaluating recruitment, spatial distribution, immune-cell interactions, and toxicity. Because TAMs are shaped by organ environment and treatment pressure, model selection can strongly influence conclusions.

### 8.1 *In vitro* models

Common *in vitro* models include RAW264.7 cells, THP-1-derived macrophages, bone-marrow-derived macrophages (BMDMs), human peripheral-blood-monocyte-derived macrophages, and tumor-cell-macrophage co-culture systems. Compared with single cell lines, BMDMs and human macrophages are closer to *in vivo* states. Three-dimensional tumor spheroids, tumor organoids, and microfluidic models better simulate hypoxia, matrix barriers, and cell-cell communication. *In vitro* studies should evaluate nanoparticle uptake, cytotoxicity, endosomal escape, cytokine release, phagocytic function, and T-cell co-culture responses.

Human macrophage models are particularly important for translational relevance because receptor expression and cytokine responses differ between mouse and human myeloid cells. However, human monocyte-derived macrophages may not fully represent tissue-resident TAMs. Therefore, *in vitro* studies should avoid overgeneralization and should use multiple models when possible.

### 8.2 *In vivo* tumor models

*In vivo* models include subcutaneous xenografts or syngeneic tumors, orthotopic models, spontaneous tumor models, metastatic models, and humanized mouse models. Subcutaneous models are convenient for preliminary efficacy and imaging but differ from clinical solid tumors in immune microenvironment. Orthotopic models better reproduce organ-specific microenvironments. Spontaneous tumors and genetically engineered mouse models more closely mimic tumorigenesis. Humanized models help evaluate human TAM targets and immunotherapy combinations. Results from different models are not interchangeable, so model selection and limitations should be stated explicitly.

Syngeneic mouse models remain useful for studying immunotherapy, but many grow rapidly and show immune landscapes that differ from human tumors. Orthotopic pancreatic, brain, and liver tumor models may better capture TAM origin and spatial organization. Metastasis models are important because TAMs and macrophages contribute to pre-metastatic niches and metastatic colonization. The use of multiple complementary models can reduce the risk that nanomedicine efficacy reflects a model-specific artifact.

### 8.3 Evaluation of TAM targeting and functional reprogramming

TAM targeting can be evaluated by *in vivo* fluorescence or radiographic imaging, co-localization in tissue sections, and flow cytometric analysis of nanoparticle uptake in sorted immune-cell subsets. Phenotypic profiling can include commonly reported M1-associated markers such as CD80, CD86, MHC-II, iNOS, TNF-α, and IL-12, and M2-associated markers such as CD206, CD163, Arg-1, IL-10, and TGF-β. Methods include qPCR, Western blotting, ELISA, immunofluorescence, immunohistochemistry, and flow cytometry. Higher-level evaluation should include single-cell RNA sequencing, spatial transcriptomics, and multiplex immunofluorescence to distinguish TAM subtypes and spatial niches.

Functional evaluation is essential because marker changes may not correspond to antitumor activity. Phagocytosis assays, antigen-presentation assays, tumor-cell killing assays, cytokine profiling, and T-cell co-culture systems can reveal whether TAMs have acquired immune-stimulatory functions. When possible, sorted TAMs should be analyzed separately from other myeloid cells to avoid attributing changes in dendritic cells or monocytes to TAM reprogramming. Specifically, TAM phagocytosis assays should distinguish internalized tumor material from surface binding; antigen-presentation assays should test antigen-dependent T-cell proliferation, cytokine production, or cytotoxicity after co-culture with treated TAMs. Cargo washout and responder-cell controls help exclude direct drug effects on T cells.

### 8.4 Evaluation of antitumor immune responses

Evaluation of antitumor immune responses should include CD8^+^ T-cell infiltration, T-cell activation and exhaustion status, granzyme B, perforin, IFN-γ, Treg and MDSC proportions, dendritic-cell maturation, antigen-specific T-cell responses, and immune memory. Efficacy endpoints include tumor volume, survival, metastatic burden, recurrence rate, and response to combined ICI. Safety endpoints include body weight, complete blood counts, liver and kidney function, inflammatory cytokines, major-organ pathology, and long-term toxicity.

A coherent pharmacodynamic design should link each assay to a mechanistic question. If the cargo is a TLR agonist, cytokine kinetics and myeloid activation are central. If the cargo is an siRNA, target knockdown in TAMs must be demonstrated. If the goal is checkpoint synergy, T-cell infiltration, cytotoxicity, and durable tumor control should be assessed. This claim-evidence alignment is particularly important for review and regulatory evaluation of complex nanomedicines.

## 9 Clinical translation and current limitations

Although many preclinical studies show that TAM-targeted nanomedicines can remodel the tumor immune microenvironment and enhance immunotherapy, clinical translation remains at an early stage. A central challenge is that mouse TAMs and human TAMs differ in cellular origin, molecular markers, spatial distribution, and treatment response. Consequently, targeting strategies effective in mouse models do not automatically predict human efficacy. Key targets and regulatory pathways therefore require validation using human samples, organoids, humanized mouse models, and real clinical cohorts. As outlined in Sections 2.1, 4.3, targeting performance may also vary among lesions and treatment stages within the same patient. Receptor expression alone is therefore unlikely to predict nanocarrier delivery or reprogramming efficacy. Future development should integrate single-cell and spatial profiling, quantitative imaging, circulating biomarkers, and *ex vivo* functional assays to establish patient-specific predictors of TAM exposure and pharmacodynamic response. Companion diagnostics should be used to enrich clinical studies for tumors with high target-positive TAM burdens and defined immune-exclusion or metabolic signatures (Pittet et al., 2022; Cassetta and Pollard, 2023; Mahmoudi et al., 2023).

The functional criteria in Sections 2.2, 8.3 provide a basis for clinical pharmacodynamic assessment. TAM phagocytosis indices, antigen-presentation scores, IFN-pathway activation, CD8^+^ T-cell effector scores, and spatial proximity between TAMs and effector T cells are candidate measures that require clinical validation.

The specificity, protein-corona, and hepatic/splenic clearance issues discussed in Sections 4.3 and 7.1–7.2 must be integrated with cell-resolved exposure and safety measurements. In addition to biological delivery barriers, manufacturing and quality control are major translational bottlenecks. Particle size, polydispersity index, zeta potential, drug loading, encapsulation efficiency, release profile, ligand density, endotoxin levels, sterility, storage stability, and batch consistency can all affect efficacy and safety. For biomimetic systems, membrane-coated particles, and extracellular-vesicle preparations, scalable manufacturing and standardized control under GMP conditions are especially challenging.

Translation is also constrained by cost of goods and by the mismatch between preclinical models and human disease. Complex multicomponent formulations may require specialized raw materials, aseptic manufacturing, high-resolution analytical methods, cold-chain storage, and platform-specific release testing, all of which may undermine scalability even when efficacy is strong. Development programs should define a clinically justified minimum design, establish GMP-compatible critical quality attributes and cost-of-goods targets early, and benchmark the platform against simpler formulations. Rodent studies should be complemented, when appropriate, by human tumor slices, patient-derived organoids, humanized models, and selected large-animal studies that better capture biodistribution, immunotoxicity, administration, and repeated-dose behavior. Early-phase trials should incorporate pharmacokinetic, cell-resolved biodistribution, immune pharmacodynamic, and biomarker endpoints to determine whether preclinical TAM reprogramming is reproduced in patients (Joyce et al., 2024).

The clinical use of TAM-targeted nanomedicine with immunotherapy must move beyond proof-of-concept antitumor efficacy. It should define appropriate patient populations and dosing regimens. Potentially responsive patients may include those with high TAM infiltration, strong M2-like immunosuppressive signatures, CD8^+^ T-cell exclusion, or resistance to PD-1/PD-L1 therapy. Future clinical trials should combine histopathology, spatial omics, blood biomarkers, and imaging to stratify patients and should include TAM state changes as pharmacodynamic endpoints. Consistent with the translational roadmap in Figure 5, this approach can help move TAM-targeted nanodelivery from preclinical promise toward testable, scalable, and translatable therapeutic strategies.

**FIGURE 5:**
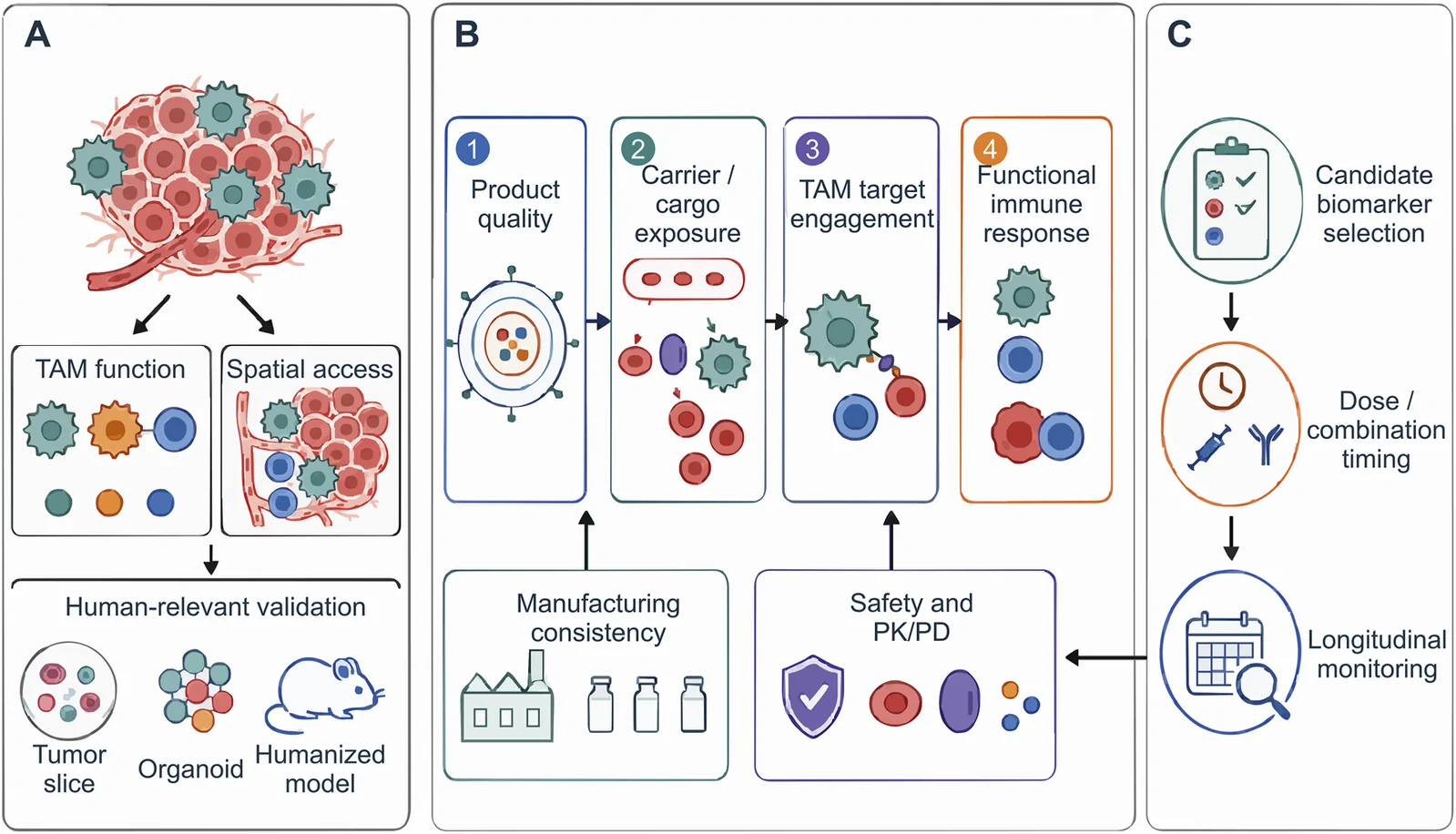
Translational roadmap for TAM-directed nanomedicine. **(A)** Human disease characterization links the relevant TAM functional state and spatial access to human-relevant validation. **(B)** Quantitative development connects product quality, carrier and cargo exposure, TAM target engagement, and functional immune responses, supported by manufacturing consistency, safety, and PK/PD analysis. **(C)** Clinical evaluation integrates candidate biomarkers, dose and combination timing, and longitudinal monitoring, with feedback to development. This roadmap depicts proposed evidence requirements rather than established clinical efficacy. PK, pharmacokinetics; PD, pharmacodynamics.

Clinical translation also requires realistic comparison with existing modalities. TAM-targeted nanomedicines may be most useful where conventional systemic immunomodulation is limited by toxicity or where local immune remodeling is needed before checkpoint blockade. The formulation should therefore justify its complexity by measurable benefits, such as increased tumor exposure, reduced systemic cytokines, improved T-cell infiltration, or superior combination efficacy. Without such evidence, additional targeting ligands or responsive modules may increase regulatory burden without improving clinical value.

## 10 Discussion and future perspectives

The future development of TAM-targeted nanoimmunotherapy should shift from broad TAM depletion toward more precise TAM reprogramming. Macrophages are important for tissue homeostasis, injury repair, and host defense, so complete depletion may disrupt normal immune balance and remove TAM subtypes with potential antitumor activity. A more rational strategy is to identify the functional TAM populations that drive immunosuppression, metastasis, and treatment resistance and then use nanodelivery systems to achieve local, reversible, and monitorable functional remodeling. As single-cell and spatial omics mature, treatment-relevant TAM subtypes will be defined more accurately, providing a stronger biological basis for target selection, patient stratification, and efficacy prediction.

TAM-targeted nanoplatforms also need to evolve from conventional single-function carriers toward programmable nanomedicines. An ideal nanoplatform should combine blood stability, tumor accumulation, TAM recognition, microenvironmental response, intracellular release, imaging-based monitoring, and safe degradation. However, multifunctionality does not mean that greater structural complexity is always better. For clinically oriented nanomedicines, every design layer should correspond to a clear and measurable pharmacological benefit, such as improved tumor exposure, enhanced TAM uptake, optimized release, reduced systemic toxicity, or better combination with immunotherapy. Complexity without measurable value increases manufacturing difficulty and regulatory burden.

The functional biomarkers discussed in Sections 6 and 8.3 require validation for predicting clinical benefit, particularly in combinations with immune checkpoint inhibitors. Linking TAM state changes to CD8^+^ T-cell function and durable tumor control remains the key pharmacodynamic question.

PK/PD modeling and companion diagnostics will directly influence the quality of clinical translation. Development programs need quantitative relationships among dose, systemic exposure, tumor accumulation, TAM uptake, cytokine release, T-cell infiltration, and tumor inhibition early in development. Imaging tracers, liquid biopsy, and spatial pathology can serve as companion diagnostic tools to identify patients with high TAM infiltration, prominent myeloid immunosuppression, or resistance to PD-1/PD-L1 therapy, and to dynamically monitor TAM state and immune-microenvironment changes during treatment.

Combination therapy remains a major opportunity for TAM reprogramming. TAM-targeted nanomedicine may be combined with checkpoint blockade, radiotherapy, chemotherapy, photodynamic or photothermal therapy, and cancer vaccines. Beyond stronger antitumor effects, clinically relevant questions include which patients are suitable for combination treatment, when each component should be administered, and how immune efficacy can be increased without excessive inflammation or systemic toxicity. Only when target selection, delivery design, functional evaluation, patient stratification, and dosing strategy form a closed translational loop can TAM-targeted nanoimmunotherapy move from proof of concept to precision immunotherapy.

Several technical directions are particularly important. First, TAM-targeted systems should incorporate human-relevant validation earlier, including human tumor slices, patient-derived organoids, and clinical samples. Second, spatially resolved analysis should be used to identify whether nanomedicines reach the TAM subtypes located at immune-exclusion interfaces, hypoxic niches, or metastatic fronts. Third, scalable manufacturing should be considered during design rather than after proof of efficacy. Finally, safety testing should include immune activation, repeated dosing, long-term material retention, and interaction with checkpoint blockade.

## 11 Conclusion

TAMs are key cellular components of the immunosuppressive tumor microenvironment and important pharmacological targets for cancer immunotherapy. Their high plasticity makes TAM reprogramming a feasible strategy to enhance antitumor immune responses.

Smart nanodelivery systems improve drug stability, tumor accumulation, TAM targeting, and stimuli-responsive release, providing more controllable platforms for TLR and STING agonists, small-molecule pathway modulators, nucleic acid therapeutics, cytokines, and macrophage immune-checkpoint modulators.

The pharmacodynamic significance of TAM reprogramming lies in relieving immunosuppression, enhancing antigen presentation, promoting CD8^+^ T-cell infiltration and cytotoxic killing, converting cold tumors into hot tumors, and improving immune checkpoint blockade such as anti-PD-1 or anti-PD-L1 therapy.

Clinical development requires durable functional responses, human-relevant validation, safe and reproducible manufacturing, and PK/PD-guided patient selection and dosing.
